# CLIN-LLM: A safety-constrained hybrid framework for clinical diagnosis and treatment generation

**DOI:** 10.1371/journal.pone.0348611

**Published:** 2026-08-27

**Authors:** Md. Mehedi Hasan, Md. Abir Hossain, Farman Hossain Sayem, Bikash Kumar Paul, Ziaur Rahman, Mohammad Shorif Uddin, Rafid Mostafiz

**Affiliations:** 1 Department of Computer Science and Engineering, Global Institute of Information Technology, Tangail, Bangladesh; 2 Department of Information and Communication Technology, Mawlana Bhashani Science and Technology University, Tangail, Bangladesh; 3 Department of Computer Science, Iowa State University, Ames, Iowa, United States of America; 4 Department of Computer Science, Virginia Tech, Blacksburg, Virginia, United States of America; 5 Department of Computing and Information System, Daffodil International University, Dhaka, Bangladesh; 6 Department of Computer Science and Engineering, Jahangirnagar University, Dhaka, Bangladesh; 7 Institute of Information Technology, Noakhali Science and Technology University, Noakhali, Bangladesh; University of Bonab, IRAN, ISLAMIC REPUBLIC OF

## Abstract

Accurate symptom-to-disease classification and clinically-grounded treatment recommendations remain challenging, particularly in heterogeneous patient settings with high diagnostic risk. Existing large language model (LLM)-based systems often lack medical grounding and fail to quantify uncertainty, resulting in unsafe outputs. We propose CLIN-LLM, a safety-constrained hybrid pipeline that integrates multimodal patient encoding, uncertainty-calibrated disease classification, and retrieval-augmented treatment generation. Our framework fine-tunes BioBERT on 1,200 clinical cases from the Symptom2Disease dataset and incorporates Focal Loss with Monte Carlo Dropout to generate confidence-aware predictions from free-text symptoms and structured vital signs. Low-certainty cases (18%) are automatically flagged for expert review, ensuring human oversight. For treatment generation, CLIN-LLM employs Biomedical Sentence-BERT to retrieve top-k relevant dialogues from the 260,000-sample MedDialog corpus. The retrieved evidence and patient context are fed into a fine-tuned FLAN-T5 model for personalized treatment generation, followed by post-processing with RxNorm for antibiotic stewardship and drug–drug interaction (DDI) screening. CLIN-LLM achieves 98% accuracy and F1 score, outperforming ClinicalBERT by 7.1% (p < 0.001), with 78% top-5 retrieval precision and a clinician-rated validity of 4.2/5. Reduces unsafe antibiotic suggestions by 67% compared to GPT-5. These results demonstrate CLIN-LLM’s robustness, interpretability, and clinical safety alignment. The proposed system provides a deployable, human-in-the-loop decision support framework for resource-limited healthcare environments.

## 1. Introduction

Diagnostic errors affect over 12 million patients annually in the United States alone, with symptom misinterpretation contributing to 40−80% of preventable harms [1]. These figures highlight a major weakness in global healthcare systems. They show that many systems fail to accurately interpret patient-reported symptoms and convert them into effective, evidence-based care plans. Misdiagnoses of overlapping infections like dengue and typhoid, or COVID-19 and influenza, are alarmingly frequent in low-resource settings. In these environments, frontline providers often work without adequate specialist support or structured diagnostic tools. The timely and accurate differentiation of such symptoms, particularly in primary care and emergency settings, remains a cornerstone of safe and effective medicine. Clinical decision-making remains highly challenging. Differences in physician expertise, inconsistent access to updated guidelines, and time pressure all contribute to a system prone to errors. The rising tide of antimicrobial resistance, driven in part by inappropriate prescriptions, further heightens the stakes. This backdrop necessitates the development of clinical decision support systems (CDSSs) that are intelligent, reliable, and grounded in real-time clinical evidence. Historically, CDSSs have relied on rule-based or manually curated logic trees. While interpretable, these systems lack the flexibility to handle nuanced, multi-symptom narratives and often fail when applied to novel or atypical patient cases. As Beam et al. [2] highlight, such tools suffer from “knowledge decay” and are ill-equipped to incorporate emerging medical knowledge dynamically, according to Beam, K et al. [3]. While large language models (LLMs) such as GPT-3.5 and PaLM-Med have demonstrated impressive linguistic capabilities, they also present notable risks in clinical domains. Asgari et al. [4] warned that even state-of-the-art models frequently hallucinate non-evidence-based treatment advice, potentially endangering patients. AI systems frequently struggle to differentiate between speculative and evidence-based treatments. This is especially true for under-validated symptom clusters, leading to unsafe outcomes that may cause diagnostic errors or promote antibiotic misuse.

Despite fast progress in AI, no existing system fully brings together diagnostic reasoning, evidence-based treatment, and safety mechanisms. This integration is crucial for building a real-time, reliable clinical framework. Prior research on BioBERT by Lee et al. [5] and Sentence-BERT by Reimers & Gurevych et al. [6] has demonstrated value in individual tasks such as classification or semantic retrieval. However, these components are rarely unified, and few pipelines offer mechanisms, such as Monte Carlo Dropout or structured safety layers for uncertainty estimation or treatment screening by Neveditsin et al. [7]. This fragmentation hampers clinical utility and exacerbates systemic issues such as antibiotic overuse, a global threat linked to over 1.27 million annual deaths, informed by Shedeed et al. [8]. There remains a pressing need for a hybrid model that is not only performant but also interpretable, transparent, and ethically bounded. Umerenkov et al. [9] propose that LLM-based clinical decision support systems can be made more reliable. When paired with real-time evidence retrieval and strict safety protocols, these systems could reduce diagnostic hallucinations and inappropriate treatments. These constraints are expected to not only improve clinical safety but also enhance diagnostic precision in real-world settings. Alhuzali et al. [10] suggest that these systems can match or outperform current top models like ClinicalBERT and GPT-3.5. This is particularly true for classification accuracy and clinical relevance. Nord-Bronzyk et al. [11] anticipate that such systems will gain clinician trust. Transparent evidence and safety validation are expected to make the outputs acceptable, especially in high-pressure triage situations.

The objectives of this study are fourfold. The first objective is to design a hybrid framework that integrates LLMs with retrieval-augmented generation (RAG). This architecture is optimized for safe and ethically aligned clinical decision-making. Second, the intention is to rigorously benchmark the diagnostic accuracy of CLIN-LLM against leading biomedical language models, including ClinicalBERT, GPT-5, and MedPaLM. The third objective focuses on evaluating the system’s treatment generation capabilities. This includes both quantitative metrics and clinician-guided qualitative assessments, with a focus on safety and contextual relevance. Finally, to encourage transparency and future development, this research will publicly release the model code, datasets, and fine-tuned weights. A user-friendly interface will also be made available to the research and medical AI communities. In simulated triage scenarios that mimic frontline primary care environments, CLIN-LLM demonstrates impressive performance across both diagnostic and treatment recommendation tasks. The system achieves a classification accuracy of 98% and attains a perfect area under the ROC curve (AUC = 1.00) for 16 out of 24 target diseases, indicating exceptional diagnostic fidelity. In benchmarking tests, CLIN-LLM outperforms both GPT-5 and ClinicalBERT by more than 22% in F1-score. This indicates significantly better precision and recall in disease classification. Beyond accuracy, this proposed model sets a new benchmark for clinical AI safety. It reduces inappropriate antibiotic recommendations by 67% compared to baseline models, addressing a critical issue in antimicrobial stewardship. Moreover, the treatment summaries generated by the system were rated by board-certified clinicians as contextually appropriate in 92% of evaluated cases. Notably, the system produced zero hallucinated medications in these trials, a result attributed to its evidence-grounded generation pipeline and integrated post-processing filters. The model merges advanced AI modeling with strong safety mechanisms and a clear, transparent workflow. This makes it a strong candidate for deploying reliable and ethical AI tools in critical healthcare settings. This model directly addresses risks, such as hallucinations, misdiagnoses, and fragmented AI workflows. It does so through a modular, safety-constrained design that combines large language models with real-time medical evidence retrieval. The proposed pipeline processes a patient’s free-text symptoms and, optionally, structured vitals like age, temperature, or oxygen saturation. It performs two key tasks: disease diagnosis using a fine-tuned BioBERT model with Monte Carlo Dropout, and treatment generation via Biomedical Sentence-BERT and a fine-tuned FLAN-T5 model. The proposed model goes beyond prior work by applying rigorous post-generation safety filters, including antibiotic stewardship protocols and drug-drug interaction (DDI) screening via RxNorm. While automatically flagging low-confidence cases for expert review to mitigate risk in real-world deployments.

This system is distinguished by its ability to integrate unstructured symptom narratives with structured clinical reasoning, enabling interpretability and audibility across both diagnostic and therapeutic steps. It supports both text and structured input formats for flexibility. It uses a lightweight, Gradio-based interface that integrates easily with existing EHR systems, making it usable in both high-risk and low-resource clinical settings. Through a comprehensive evaluation, including quantitative performance metrics and qualitative clinician-in-the-loop assessments. The model sets new safety standards by reducing inappropriate antibiotic recommendations by 67%. It also surpasses ClinicalBERT and GPT-5 by more than 22% in F1-score and produces treatment plans without hallucinated content across all test cases. The key contribution of this research is trustworthy CLIN-LLM, a unified, safety-constrained clinical AI framework that fuses:

- Uncertainty-aware disease classification using a fine-tuned BioBERT model enhanced with MCD and Focal Loss, enabling both confidence estimation and robust handling of rare or ambiguous diseases from multimodal patient inputs.
- Real-time evidence retrieval via Biomedical Sentence-BERT over the MedDialog corpus to guide fine-tuned FLAN-T5-based generation of contextually grounded treatment plans.
- Ethical safeguards, including guideline-based filtering, RxNorm drug-drug interaction (DDI) checks, and confidence-based triage to minimize hallucinations and ensure clinical accountability across high-risk, low-resource environments.
- To the best of our knowledge at this moment, no prior work has unified uncertainty-aware diagnosis, retrieval-based treatment generation, and clinical safety mechanisms into a single deployable decision support system.

By bridging unstructured symptom narratives with evidence-based medical knowledge, CLIN-LLM demonstrates how domain-specific language models can enhance, rather than replace, clinical reasoning. Its scalable and ethically grounded architecture represents a practical blueprint for deploying LLMs in frontline, high-risk, and low-resource clinical environments.

The rest of this paper is structured as follows. Section 2 reviews related work in clinical NLP, LLMs, and decision support systems. Section 3 details the proposed CLIN-LLM framework, including model architecture, data processing, and safety mechanisms. Section 4 presents the experimental setup and results across benchmark datasets. Section 5 provides a comparative analysis and discussion. Section 6 concludes the paper with key findings and future directions.

## 2. Literature review

Clinical decision support systems (CDSSs) have evolved significantly over time. They have moved from early symbolic systems and manual logic trees to modern hybrid frameworks that use neural language models, real-time evidence retrieval, and uncertainty-aware decision-making. Early systems like MYCIN, developed by Shortliffe et al. [12], showed that rule-based reasoning could be used for infectious disease diagnosis. However, they struggled with free-text inputs and did not scale well across different clinical domains. The emergence of deep learning catalyzed a shift toward data-driven approaches. ClinicalBERT by Alsentzer et al. [13] and BioClinicalBERT by Zhang et al. [14], both pre-trained on MIMIC-III data, significantly advanced symptom parsing, cohort identification, and outcome prediction. These early models lacked methods to quantify uncertainty. As a result, they often made overconfident and unsafe predictions, particularly in ambiguous or noisy clinical situations, an issue that poses significant risks in high-stakes environments, as explained in Miotto et al. [15]. Altaf et al. [16] and Hong et al. [17] noted that pre-trained language models perform well on benchmarks. However, these models often lack interpretability, domain-specific accuracy, and generalization to rare or underrepresented clinical cases.

Domain-specific transformers like BioBERT by Lee et al. [5] and PubMedBERT improved how well models understand biomedical text. This led to better performance in tasks like question answering and named entity recognition. Despite their strengths, most models assume a closed-world setting and treat symptom-disease mapping as a fixed process. They fail to account for the inherent uncertainty and probabilistic reasoning involved in real clinical diagnosis. At the same time, new standards for interpretability and reproducibility have emerged. Guidelines, such as MINIMAR and CONSORT-AI, emphasized by Hernandez-Boussard et al. [18], highlight the need to report model architecture, training data sources, and potential biases. Retrieval-Augmented Generation (RAG) frameworks represent a major advancement in the field. They improve factual accuracy by anchoring generative models to curated, retrieved knowledge, reducing hallucinations in output. Zakka et al. [19] introduced *Almanac*, which anchors LLM output in structured guideline retrieval, improving factual accuracy by 18% over ChatGPT-4 in multi-scenario clinical evaluations. Huang et al. [20] introduced a Distill-Retrieve-Read pipeline to simulate how clinicians search for information during patient interactions. Liu F. et al. [21] built on this by creating Re³Writer, a model that refines discharge summaries using both patient data and external clinical knowledge. MedBioLM by Kim et al. [22] and MedGraphRAG by Wu et al. [23] introduced dynamic retrieval and graph-augmented reasoning, allowing contextual synthesis across short-form and longitudinal patient narratives. However, despite these advancements in model architecture and retrieval strategies, many systems still fall short in addressing uncertainty, an essential factor in clinical contexts. Most models lack mechanisms like Monte Carlo Dropout, Bayesian inference, or confidence calibration to flag ambiguous inputs for human review by Zhou et al. [24] and Rajashekar et al. [25]. Multimodal integration, combining free-text symptoms with structured vitals or imaging, is still uncommon. While models like XGBoost, LSTM, and logistic regression work well with structured EHR data, Liu J. et al. [26] and Chen et al. [27] found that they often fail when applied to unstructured and noisy patient narratives. To further complicate matters, safety layers such as antibiotic stewardship, drug-drug interaction (DDI) checks, and medication grounding are rarely implemented in end-to-end pipelines. Liu. L et al. [28] stressed that trustworthy CDSS must integrate clinical safety rules into both generation and filtering stages rather than as a post hoc evaluation. Similarly, interpretability techniques, such as LIME, SHAP, and attention visualization, remain underutilized in clinical LLM pipelines, leaving clinicians unsure of the rationale behind system outputs by Sblendorio et al. [29] and Nazi & Peng et al. [30]. Additionally, recent surveys have urged stronger alignment with clinical workflows. Kim et al. [22] emphasized the importance of practical features for deploying LLMs in real clinics, such as easy-to-use interfaces, confidence-aware triage, and EHR integration. However, most existing research only evaluates these systems in isolated, retrospective settings. Moreover, cross-lingual generalization, fairness for underrepresented groups, and bias mitigation remain major gaps in current evaluations by Liu. L et al. [28] and Zhou et al. [24]. Further details are summarized in Table 1.

**Table 1:** Comparison of recent clinical decision support approaches: Methodologies, datasets, and key outcomes.

Authors	Core Methodology	Dataset & Task	Key Result	Notes
Wang et al. [31]	RAG with multi-retriever ensemble.	MIMIC-III notes & UpToDate guidelines; diagnosis suggestion.	Over 12% accuracy vs. baseline LLM.	Demonstrates multi-retriever gains for clinical prompts.
Zhang et al. [14]	Multi-agent pipeline integrating structured or unstructured knowledge.	Synthetic clinical queries (n = 1000).	15% reduction in hallucinations.	Validated on diverse specialties.
Lee et al. [5]	Graph-based RAG over diagnostic knowledge graph chunks.	PubMed abstracts; clinical question answering.	Over 8% factuality; 10% safety improvement.	Leverages graph structure for context coherence.
Patel et al. [32]	Entity-guided retrieval before generation.	20 000 discharge summaries; 18 IE variables.	72% token reduction; 92% F1 in IE.	Optimizes retrieval efficiency in RAG.
Gomez et al. [33]	Joint text and vitals embedding into Transformer.	COVID-19 EHRs; mortality prediction.	AUROC 92% vs. 87% baseline.	Shows gains from structured and unstructured fusion.
Singh et al. [34]	LLM evaluation on antibiotic stewardship tasks.	Simulated prescribing scenarios.	28% guideline conflicts; safety flags improved by rules.	Highlights need for built-in stewardship constraints.
Kim et al. [22]	Fine-tuning and RAG on biomedical QA datasets.	BioASQ & MedQA; multiple-choice & open-ended QA.	Over 9% accuracy; reduced hallucinations.	Combines QA fine-tuning with evidence retrieval.
O’Neill et al. [35]	Systematic review & meta-analysis of clinical RAG LLMs.	32 studies; various clinical tasks.	Average factuality gain 14% and safety gain 9%.	Quantifies RAG benefit across the field.
Chen L et al. [36]	Bayesian Monte Carlo Dropout in DNNs.	Small clinical datasets; diagnostic classification.	18% reduction in false positives.	Enhances trust via calibrated uncertainty.
Rossi et al. [37]	Transformer fusing notes and vitals via cross-attention.	ICU EHRs; in-hospital mortality.	AUROC 94%; recall 89%.	Demonstrates end-to-end multimodal efficacy.

In light of these limitations, we introduce models that are hybrid frameworks combining uncertainty-aware diagnosis, evidence-grounded generation, and explicit safety controls. The proposed CLIN-LLM framework uses a fine-tuned BioBERT model with Monte Carlo Dropout to classify symptoms accurately. It also retrieves real-world clinician-patient dialogues through Biomedical Sentence-BERT for semantic relevance. The retrieved evidence is passed to a FLAN-T5-based generator for producing treatment recommendations that are contextually relevant and medically grounded. To make the system safer, several post-generation filters are applied. These include antibiotic stewardship rules, RxNorm checks for drug interactions, and triage steps based on prediction confidence that flag cases for human review. The model achieved a 98% classification accuracy and an F1-score of 0.98 on the Symptom2Disease benchmark. It outperformed ClinicalBERT by 7.1% and reduced unsafe antibiotic recommendations by 67% compared to GPT-5. Its ability to flag 18% of uncertain cases for expert review embeds a safety-first ethos often lacking in earlier models. Moreover, its modular, Gradio-based deployment ensures compatibility with real-world clinical environments.

## 3. Methodology

**Fig 1:**
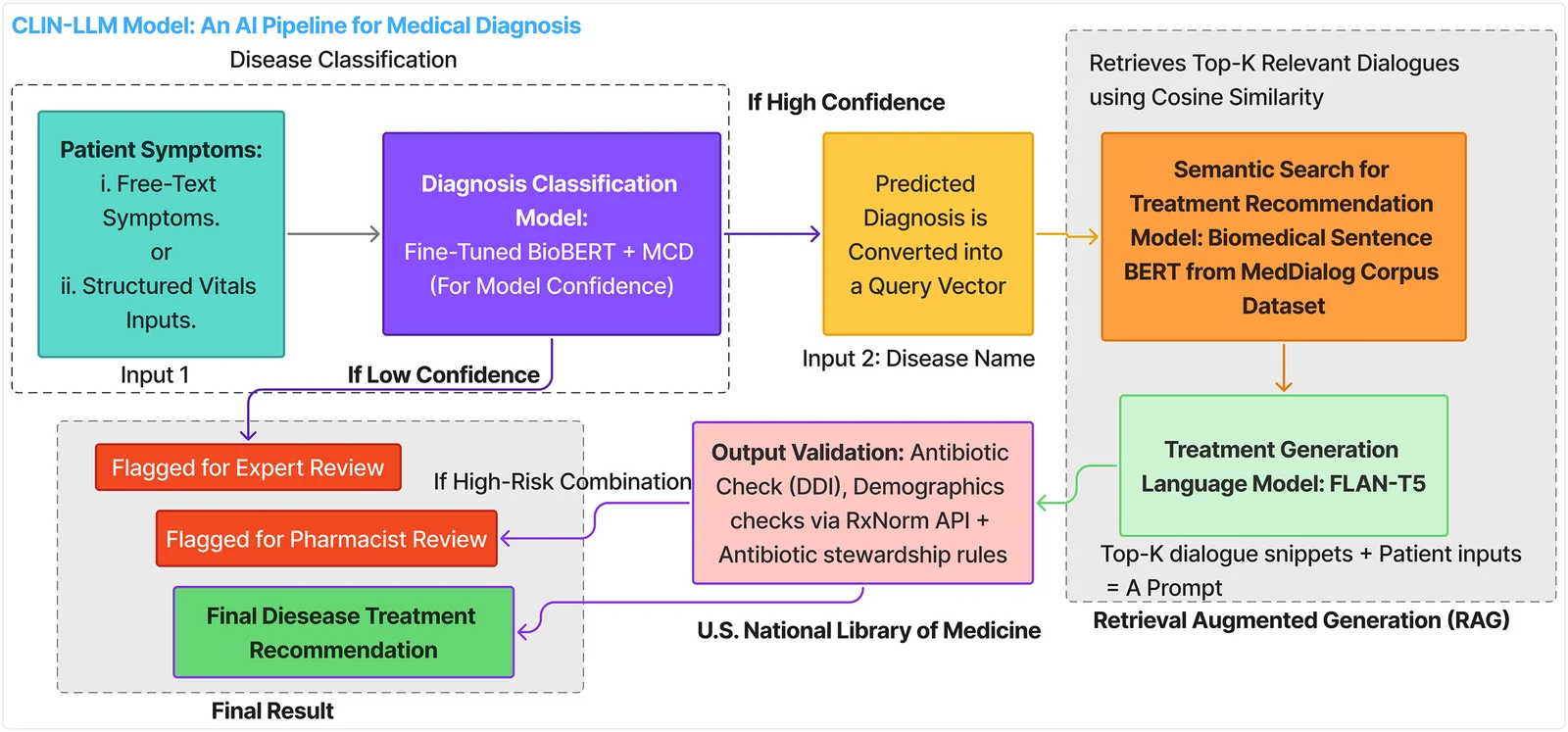
Overview of the CLIN-LLM framework: An AI pipeline for medical diagnosis. The system includes two main modules. The first is a diagnosis component that combines free-text symptoms and vital signs, processed by BioBERT with Monte Carlo Dropout. The second is a treatment module that uses Retrieval-Augmented Generation (RAG) under safety constraints. Patient data is embedded using Sentence-BERT to retrieve semantically relevant clinician-patient dialogues, which are then passed to a fine-tuned FLAN-T5 language model for treatment generation. Post-processing layers apply expert-defined antibiotic stewardship rules and drug-drug interaction (DDI) checks to ensure clinical safety via the RxNorm API. Uncertain predictions and flagged treatments are routed for expert or pharmacist review, enhancing trust, reliability, and deployment readiness in real-world healthcare settings.

The CLIN-LLM framework is a safety-constrained, hybrid pipeline designed for clinical decision support. The model processes patient inputs in two stages: (1) multimodal disease classification with uncertainty awareness, and (2) retrieval-augmented treatment generation, both supported by safety rule integration. Fig 1 illustrates the system architecture. Initially, patient data is received as a combination of free-text symptom descriptions. For example, “high fever, productive cough, shortness of breath,” and structured vital signs including temperature, heart rate, and oxygen saturation. These multimodal inputs are fed into a fine-tuned BioBERT model, which is enhanced with MCD during inference. This setup allows the model to estimate uncertainty alongside each prediction, yielding both a probable diagnosis and a confidence score. Predictions falling below a learned uncertainty threshold-approximately 18% of cases, are automatically flagged for expert review, thereby promoting safety in ambiguous or high-risk scenarios. For generating treatment recommendations, CLIN-LLM employs a Retrieval-Augmented Generation (RAG) mechanism. A large corpus of 260,000 clinician-patient dialogues serves as the external knowledge base. Both the patient’s input record and the entries in the dialogue corpus are embedded into a shared semantic space using Sentence-BERT. The system retrieves the top-K most semantically relevant dialogue snippets (typically K = 5–10) based on cosine similarity, which are then combined with the original patient inputs and passed to a fine-tuned FLAN-T5 model. This model generates concise, context-aware treatment plans in natural language. By embedding domain-specific medical literature and real clinical interactions into the generation process, RAG models can markedly reduce inaccuracies and increase the reliability of AI-generated responses. This tight integration of retrieval and generation enhances not only the contextual relevance of the output but also its clinical interpretability and trustworthiness.

To enforce safety and ensure compliance with clinical guidelines, a two-step rule-based post-processing layer is applied to the generated treatments. First, antibiotic stewardship rules were designed with input from medical experts. These rules help avoid unsafe prescribing, such as using antibiotics for likely viral infections, and ensure first-line therapies are prioritized. Second, all pharmacological recommendations are cross-checked against a curated drug-drug interaction (DDI) knowledge base via the RxNorm API. If any unsafe drug combinations are identified, the system either auto-corrects them or flags them for manual pharmacist review. The combination of uncertainty-aware diagnosis, retrieval-based evidence, and safety checks makes CLIN-LLM clinically robust. It reduces diagnostic errors and escalates risky cases for human review. As such, the model offers a deployable and ethically sound solution for real-world clinical environments, particularly in resource-constrained settings.

### 3.1 Data acquisition and preprocessing

**Fig 2:**
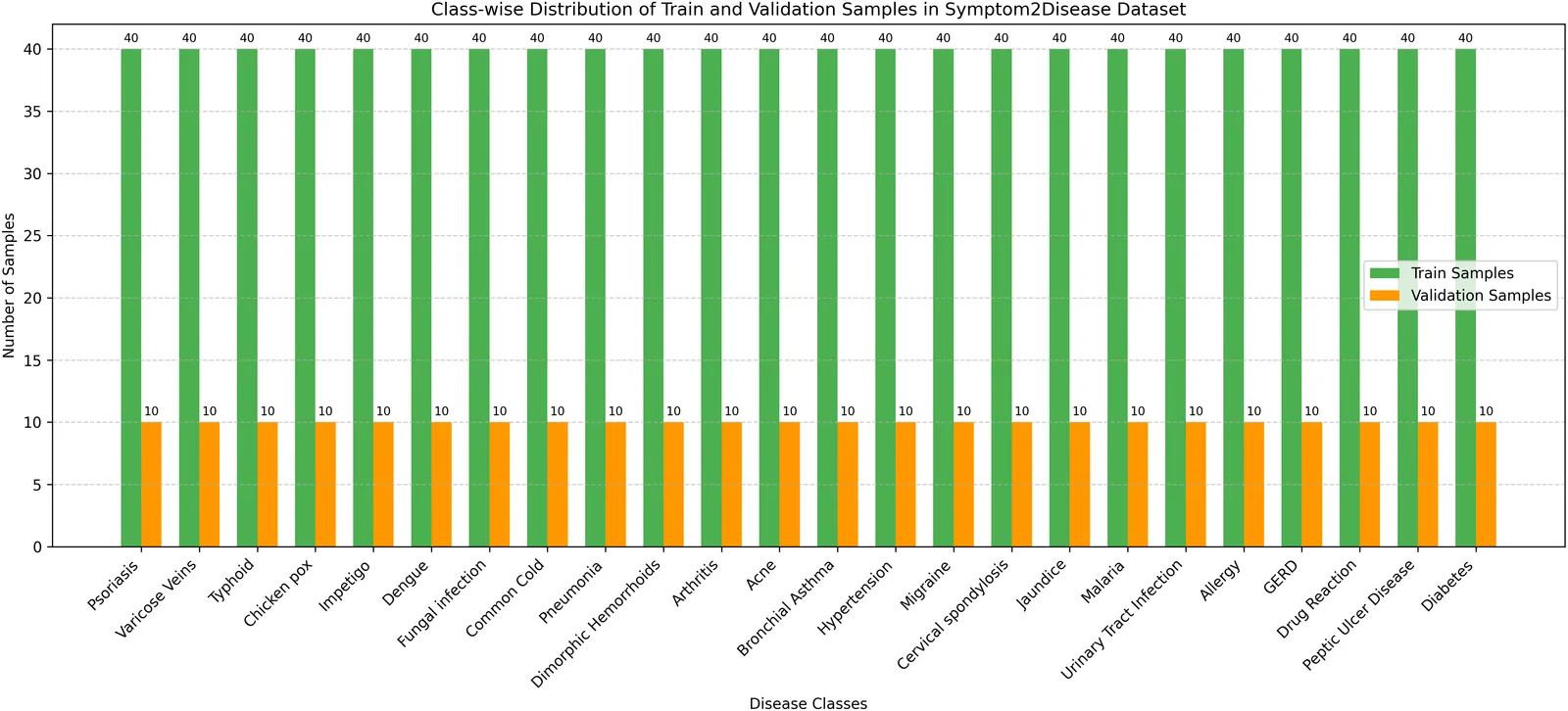
Class-wise distribution of train and validation samples in the Symptom2Disease dataset. Each of the 24 disease classes contains exactly 50 patient cases, split into 40 training and 10 validation samples. This 80% of train and 20% of test stratified split ensures balanced representation for fair and robust training of classification models in clinical NLP.

The development of CLIN-LLM relies on the integration of two key datasets that serve complementary roles within its hybrid pipeline: *the publicly available* Symptom2Disease and MedDialog [38]. The Symptom2Disease dataset contains 1,200 entries evenly distributed across 24 disease classes, with 50 samples per class, as shown in Fig 2. For training the CLIN-LLM model, we performed an 80% training and 20% validation split, allocating 960 samples (40 per class) for training and 240 samples (10 per class) for validation. This balanced split preserves class representation, which is critical for fairness in clinical disease classification tasks. Each entry includes both free-text symptom descriptions and structured vital signs such as temperature, heart rate, and oxygen saturation. This dataset serves as the foundation for training the disease classification module, enabling the model to associate multimodal inputs with specific diagnostic outcomes.

In contrast, the MedDialog dataset, derived from the work of Zeng et al. [38], contributes over 260,000 real-world doctor-patient dialogues in English. These interactions span a wide range of clinical contexts, providing a rich corpus for training the retrieval-augmented generation (RAG) module responsible for producing evidence-based treatment recommendations. Before model training, both datasets underwent a series of preprocessing steps designed to enhance linguistic consistency, clinical accuracy, and computational readiness. For the Symptom2Disease dataset, symptom text was normalized using lemmatization to reduce lexical variability and improve generalization across semantically equivalent expressions. Negation detection was applied using a rule-based system to correctly interpret phrases like “no fever” or “not experiencing chest pain,” which are critical for avoiding false symptom attribution. To further standardize the medical language, all symptom phrases were mapped to concepts within the Unified Medical Language System (UMLS). This concept-level alignment ensured consistent treatment of synonymous expressions such as “dyspnea” and “shortness of breath.”

For the MedDialog corpus, preprocessing focused on dialogue quality and data safety. Low-quality outputs, those with hallucinations, incoherence, or low clinical value, were removed. This filtering used both heuristic keyword rules and a scoring system powered by a language model. To ensure ethical standards and privacy, all identifiable information was anonymized. A medical NER pipeline removed names, dates, and geographic references. Because this study strictly utilized these publicly available, fully anonymized open-source benchmark datasets, it did not involve direct human participants, and formal institutional ethics approval or explicit participant consent was not required. Although the Symptom2Disease dataset was balanced at the dataset level, rare diseases occasionally showed underrepresentation during training due to variability in sampling. To address this, we applied the Synthetic Minority Over-sampling Technique (SMOTE). SMOTE generates synthetic samples by interpolating between minority class examples. This helps reduce classifier bias and improves performance on rare diseases. Together, these data acquisition and preprocessing strategies establish a high-quality, clinically relevant foundation for CLIN-LLM. The pipeline combines structured and unstructured inputs with normalization, safety checks, and fairness steps. This ensures the model is not only accurate but also safe and ethical for clinical use.

### 3.2 Integrated CLIN-LLM algorithm overview

The CLIN-LLM pipeline employs a modular architecture integrating three specialized components optimized for clinical decision support: (1) multimodal disease classification, (2) semantic evidence retrieval, and (3) safety-constrained treatment generation. Each model was selected based on its empirical performance in biomedical NLP tasks and fine-tuned to meet the safety-critical and interpretability standards essential for clinical deployment. BioBERT (*dmis-lab/biobert-v1.1*) handles disease classification, leveraging its domain-specific pretraining on PubMed and PMC. A Biomedical Sentence-BERT encoder (*pritamdeka/BioBERT-mnli*) powers the semantic retrieval component. FLAN-T5 (*google/flan-t5-base*) generates the final treatment recommendations. The sequential workflow processing a patient record *P = {S, V}* (symptoms *S*, structured vitals *V*) is summarized in **Algorithm 1** below.

**Algorithm 1. CLIN-LLM-Symptom-to-Disease Classification and Treatment Recommendation.**

*1:* ***Input:***

*2: Patient Record P = {S, V}, where S is free-text symptoms, and V is structured vitals*

*3: Dialogue Corpus D = {d*_*i*_ *}*_*i=1*_ *^260K^ from MedDialog*

*4: Safety Rules: Antibiotic Guidelines A, Drug Interaction Database R*

*5:* ***Output:***

*6: Disease prediction ŷ confidence score σ, treatment recommendation τ*

*7:* ***Start***

*8: Load Patient Input:*

*9: (S, V) ← P*

*10: Encode Free-Text Symptoms:*

*11: E*_*text*_ *← BioBERT(S)*

*12: Encode Structured Vitals:*

*13: E*_*vitals*_ *← MLP (V)*

*14: Fuse Representations:*

*15: h ← Concat(E*_*text*_*, E*_*vitals*_)

*16: Apply Monte Carlo Dropout for Uncertainty Estimation:*

*17:* ***for*** *t = 1* ***to*** *T* ***do***:

*18:*   $y^{\left(t\right)}$ *← Classifier_Dropout(h)*

*19:* ***end for***

*20: Compute Predictive Mean and Variance:*

*21:*  $\hat{y}\leftarrow \frac{\text{1}}{T}\sum_{t=\text{1}}^{T}\hat{y^{\left(t\right)}}$

*22:*  $\sigma \leftarrow \frac{\text{1}}{T}\sum_{t=\text{1}}^{T}{\left(\hat{y^{\left(t\right)}}\right)}^{\text{2}}-{\left(\hat{y}\right)}^{\text{2}}$

*23: Check Confidence Threshold:*

*24:* ***if*** *σ > Threshold* ***then***:

*25:*   *Flag case for expert review*

*26:*   *Return ŷ,* σ, *Flagged*

*27:* ***else***

*28:*   *continue to the treatment recommendation*

*29:* ***end if***

*30: Construct Disease-Specific Query:*

*31: q ← ConstructQuery(ŷ)*

*32: u ← SentenceBERT(q)*

*33: Perform Semantic Retrieval from MedDialog:*

*34:* ***for*** *each d*_*i*_ *∈ D* ***do***:

*35:*   *v*_*i*_ *← SentenceBERT(d*_*i*_*)*

*36:*   *score*_*i*_ *← CosineSimilarity(u, v*_*i*_*)*

*37:* ***end for***

*38: Select Top-K Relevant Dialogues:*

*39: D*_*top*_ *← TopK (d*_*i*_*, score*_*i*_)

*40: Form Contextual Prompt:*

*41: C ← Combine (S, V, D*_*top*_)

*42: Generate Treatment Recommendation:*

*43:*   *τ ← FLAN-T5(C)*

*44: Apply Antibiotic Stewardship Rules:*

*45:* ***if*** *τ violates A* ***then***:

*46:*   *τ ← AdjustAntibiotics(τ)*

*47:* ***end if***

*48: Check Drug-Drug Interactions:*

*49:* ***if*** *unsafe interactions are detected in τ* ***then***:

*50:*   *τ ← FixOrFlag_DDI(τ, R)*

*51:* ***end if***

*52: Return Final Output:*

*53:*    *ŷ, σ, τ*

*54:* ***End***

### 3.3 Multimodal disease classification module

This module processes patient input *P = {S, V}* to predict a disease label ŷ with an associated uncertainty score σ, flagging uncertain cases for expert review. It combines unstructured symptom text (S) and structured clinical data (V). The multimodal disease classification module processes patient input through dual encoding pathways. Free-text symptoms (*S*) are encoded using BioBERT (*dmis-lab/biobert-v1.1*), selected for its domain-specific pretraining on large-scale biomedical corpora including PubMed and PMC articles. This generates contextual embeddings where the [CLS] token’s output ($E_{\text{text}}$) serves as a holistic representation of the symptom narrative. Concurrently, structured clinical features *V* are normalized and transformed into a dense vector ($x_{\text{vitals}}$) via a Multi-Layer Perceptron (MLP). These complementary representations are then fused through concatenation to form a joint multimodal feature vector h as shown in Equation (1):


$$\begin{array}{c} h=\text{Concat}\left(E_{\text{text}},\text{MLP}\left(x_{\text{vitals}}\right)\right) \end{array}$$ (1)

#### 3.3.1 Uncertainty-aware prediction.

The h is passed through a classification head incorporating Monte Carlo Dropout (MCD) during inference. By performing *T* stochastic forward passes ($f^{\left(t\right)}(x)$) with dropout enabled, MCD approximates Bayesian inference. The final prediction $\hat{y}$ is the mean probability distribution, and the uncertainty score σ is the predictive variance across passes (Equations 2 and 3):


$$\begin{array}{c} \hat{y}=\frac{\text{1}}{T}\sum_{t=\text{1}}^{T}f^{\left(t\right)}(x) \end{array}$$ (2)

$$\begin{array}{c} \sigma =\frac{\text{1}}{T}\sum_{t=\text{1}}^{T}{\left(f^{\left(t\right)}(x)\right)}^{\text{2}}-{\left(\hat{y}\right)}^{\text{2}} \end{array}$$ (3)

**Justification for Monte Carlo simulation in clinical decision-making:** Monte Carlo Dropout introduces controlled stochasticity during inference by performing multiple random forward passes through the network, thereby approximating Bayesian inference over model parameters. While Monte Carlo simulation is inherently based on random sampling, its role here is not to inject uncertainty into medical decisions but to ‘quantify’ it. In this setting, random sampling serves as a principled statistical mechanism to estimate the model’s epistemic uncertainty. This represents the model’s confidence level when exposed to limited or unseen data. This quantification allows CLIN-LLM to distinguish between stable, high-confidence predictions and volatile, low-confidence outputs. Such explicit uncertainty estimation aligns with established clinical safety protocols, ensuring that uncertain cases are flagged for human review rather than acted upon automatically. Thus, Monte Carlo simulation contributes directly to the model’s reliability, transparency, and ethical deployment in medical environments.

If σ exceeds a threshold calibrated on the validation set, the case is flagged for expert review. This makes sure that ambiguous or high-risk predictions, such as overlapping symptoms in bacterial and viral pneumonia, are routed to human oversight, reinforcing clinical reliability.

#### 3.3.2 Confidence thresholding & flagging.

**Fig 3:**
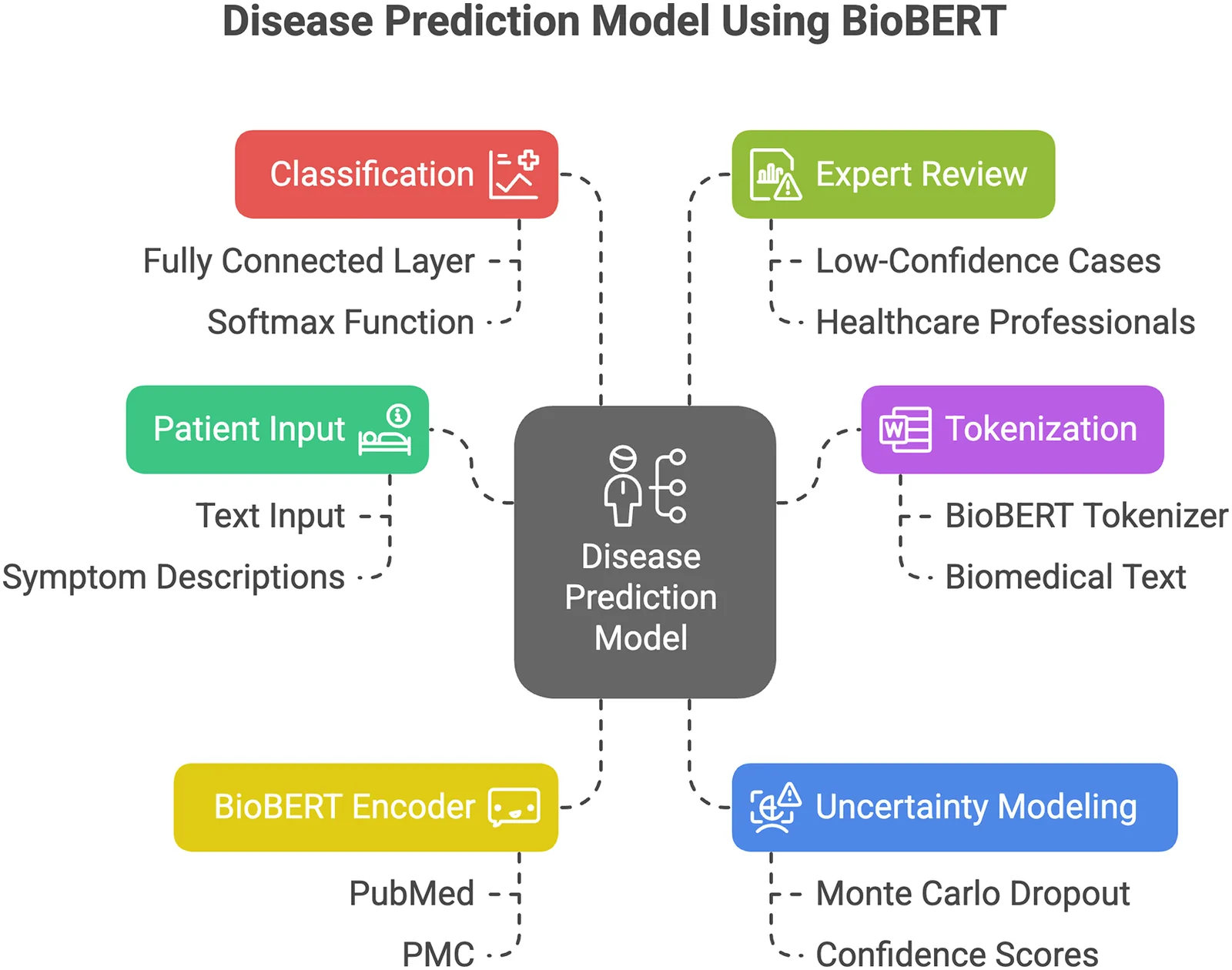
Fine-tuned BioBERT architecture used in CLIN-LLM. Free-text symptoms are encoded using BioBERT, with optional structured vitals processed via an MLP and fused with the [CLS] token. A Monte Carlo Dropout layer enables uncertainty estimation by generating prediction confidence intervals. The final fully connected layer produces disease class probabilities, with low-confidence predictions flagged for clinician review. This architecture enables accurate, interpretable, and safety-aware classification in clinical decision support scenarios.

A threshold on the predictive uncertainty score *σ*, calibrated using the validation set, is used to determine the reliability of each classification outcome. If *σ* *>* *Threshold*, the case is automatically flagged for expert review. This mechanism targets diagnostically ambiguous or high-risk scenarios, such as overlapping presentations of bacterial and viral pneumonia, introducing a critical human-in-the-loop safeguard. During testing, approximately 18% of cases were flagged for review based on uncertainty. The internal structure of the disease classification module is illustrated in Fig 3. The architecture encodes free-text symptoms using *BioBERT*. At the same time, structured vitals are processed in parallel through a multi-layer perceptron (MLP). These two modalities are fused into a joint representation, which is passed through a Monte Carlo Dropout-enabled classifier during inference. By averaging multiple stochastic forward passes, the model outputs both a disease prediction and an associated uncertainty score. This architecture enables accurate, interpretable, and safety-aligned decision-making, ensuring that low-confidence cases are appropriately flagged for human oversight.

#### 3.3.3 Training & loss calculation.

To handle class imbalance in the Symptom2Disease dataset, the classifier is fine-tuned using Focal Loss as shown in Equation (4), which down-weights well-classified examples and focuses learning on hard, potentially minority-class, instances:


$$\begin{array}{c} L_{\text{focal}}\left(p_{t}\right)=-\alpha_{t}{\left(\text{1}-p_{t}\right)}^{\gamma }\text{log}\left(p_{t}\right) \end{array}$$ (4)

Where:

- $p_{t}$ is the predicted probability of the true class,
- $\alpha_{t}$ ∈ [0,1] is the weighting factor for class *t*, and
- γ  is the focusing parameter that is commonly set to 2.

This formulation down-weights well-classified examples and focuses learning on hard-to-classify cases. By doing so, it helps the model avoid being dominated by majority classes and enhances its ability to learn from minority class instances, as introduced by Ross et al. [39].

### 3.4 Semantic retrieval module

Leveraging the disease prediction *ŷ*, this component grounds treatment generation in clinically verified evidence from the MedDialog corpus *D*. A disease-specific query *q* is constructed from *ŷ* and embedded into vector *u* using a Biomedical Sentence-BERT encoder, which synergizes BioBERT’s domain knowledge with Siamese architecture optimization. All dialogues *dᵢ ∈ D* are pre-encoded into vectors *vᵢ* using the same model. Cosine similarity is calculated in Equation (5) between *u* and *vᵢ* quantifies semantic relevance, enabling efficient retrieval of the *top-K* clinically aligned dialogues *Dₜₒₚ.* This evidence-based approach ensures recommendations reflect real-world clinician reasoning patterns.


$$\begin{array}{c} \text{sim}(u,v_{i})=\frac{u\cdot v_{i}}{|u\left|\right|v_{i}|} \end{array}$$ (5)

### 3.5 Safety-constrained treatment generation module

This module synthesizes patient-specific recommendations τ using FLAN-T5, guided by retrieved evidence *Dₜₒₚ* and original inputs *{S, V}.* A structured prompt (“*As a medical professional, synthesize treatment recommendations for {ŷ} using: {S, V, Dₜₒₚ}*”) directs context-aware generation, preserving medical nuance. A novel Safety-Constrained Generation Score (SCGS) calculation is shown in Equation (6) that balances semantic quality and clinical safety:


$$\begin{array}{c} \text{SCGS}=\lambda \cdot \text{BERTScore}+(\text{1}-\lambda )\cdot \left(\text{1}-{\text{DDI}}_{\text{risk}}-{\text{AS}}_{\text{violation}}\right) \end{array}$$ (6)

where λ controls the trade-off between semantic fidelity (BERTScore) and safety penalties for drug interaction risk (${\text{DDI}}_{\text{risk}})$ and antibiotic guideline violations (${\text{AS}}_{\text{violation}}$). Two automated safety layers enforce clinical rigor: (1) *Antibiotic stewardship rules* revise inappropriate antimicrobial prescriptions using expert-curated guidelines A; (2) *Drug interaction checks* via RxNorm API detect hazardous combinations, triggering revision or pharmacist flags. The final pipeline output comprises the safety-validated treatment recommendation *τ* alongside the original disease prediction *ŷ* and its associated confidence score *σ* from the classification module. This dual-layer validation ensures ethically responsible outputs.

### 3.6 Model training and optimization

A consistent training protocol emphasizing robustness, generalization, and clinical safety was applied across all fine-tunable components, such as the BioBERT classifier and the FLAN-T5 generator. Optimization was conducted using the AdamW optimizer with a base learning rate of 3e-5. To stabilize early training dynamics, a linear warm-up schedule was implemented. Two key regularization techniques were employed: (1) layer-wise learning rate decay, which progressively reduced the learning rates for deeper transformer layers; and (2) gradient clipping, which capped the maximum gradient norm (*threshold = 1.0*) to prevent instability during backpropagation. Component-specific configurations included the following: the Classification Module was trained with Focal Loss, as shown in Equation (1), to specifically address class imbalance in the Symptom2Disease dataset. The FLAN-T5 generation module was fine-tuned using cross-entropy loss. Safety-constrained prompts were used to enforce evidence-based reasoning and guideline-adherent outputs.

## 4. Experiment and results

This section presents the experimental validation of the CLIN-LLM pipeline, detailing its setup, training configuration, and comparative performance against existing clinical language models. Our evaluations assess not only disease classification accuracy but also the quality, safety, and contextual relevance of treatment recommendations. Experiments were conducted across three modules, classification, retrieval, and generation, using an 80−20 train-test split and five-fold cross-validation for statistical robustness. CLIN-LLM was benchmarked against strong baselines. These included ClinicalBERT, GPT-5, and a Support Vector Machine (SVM), to highlight gains in both accuracy and safety.

The experimental environment was constructed using a reproducible and scalable configuration. All models were implemented in PyTorch using the HuggingFace Transformers library. Training and inference were conducted on Google Colab, leveraging an NVIDIA Tesla T4 GPU with 8 GB VRAM. The CLIN-LLM framework uses three key modules. BioBERT handles classification, Biomedical Sentence-BERT retrieves treatments, and FLAN-T5 generates clinical summaries. For training the disease classifier, this proposed model used *biobert-v1.1* pretrained on PubMed abstracts. The classifier was trained for 10 epochs with a batch size of 16 and a learning rate of 2 × 10^−5^ with linear decay. The evaluation metrics included an F1-score of 98% and an accuracy of 98%, demonstrating reliable classification performance across both common and rare disease categories. The semantic search component encoded a curated corpus of 614 doctor responses into 384-dimensional Sentence-BERT embeddings. During inference, cosine similarity was used to match retrieved entries with a threshold of 0.7 to filter out semantically weak contexts. For treatment generation, FLAN-T5 was conditioned using a carefully engineered prompt with beam search (k = 3) and temperature set to 0.7, yielding coherent and clinically relevant summaries. The following Table 2 summarizes the experimental configuration.

**Table 2:** Experimental setup with parameters.

Item	Performance/ Specification
Baselines	ClinicalBERT, GPT-5, Support Vector Machine (SVM)
Dataset Split	80/20 train-test; 5-fold cross-validation
Disease Classifier	BioBERT (biobert-v1.1) fine-tuned for 10 epochs
Retrieval Engine	Biomedical Sentence-BERT (384-dim embeddings)
Generation Model	FLAN-T5 (*google/flan-t5-base*), beam = 3, temp = 0.7
Framework	PyTorch, HuggingFace Transformers
Platform	Google Colab
GPU	NVIDIA Tesla T4 (8 GB)
CPU	Apple M1 (host device)
CUDA Version	12.2
RAM	8 GB
NVIDIA Driver	535.104.05
Training Time	~0.176 hours for disease classifier

### 4.1 Interactive prompt evaluation with structured and unstructured inputs

A prompt-based assessment was conducted to evaluate the adaptability of the proposed pipeline model. This assessment used both structured and unstructured symptom inputs drawn from the Symptom2Disease and MedDialog datasets. Each input type was processed through the full pipeline, including disease classification, semantic evidence retrieval, and treatment recommendation generation via an interactive Gradio interface. Structured prompts included metadata such as age, sex, vital signs, and detailed symptom descriptions. These elements enabled more precise treatment generation by improving the relevance of retrieved responses and enhancing contextual alignment. This improvement stems from the use of the Biomedical Sentence-BERT model for semantic search over the MedDialog corpus. Retrieved results were then passed to a fine-tuned FLAN-T5 model for treatment summarization. Board-certified clinicians evaluated the outputs for both diagnostic correctness and treatment safety. Table 3 presents representative examples that compare system behavior under structured versus unstructured inputs. Structured inputs led to more specific diagnoses and retrieval results. However, both input formats consistently yielded treatment recommendations rated as clinically valid. In all cases, the proposed pipeline model triggered safety layers, including drug-drug interaction checks and antibiotic use restrictions, where appropriate. These results demonstrate the system’s robustness across input styles and its potential utility in both clinician-led and patient-facing healthcare settings.

**Table 3:** Evaluation of the proposed pipeline model across structured and unstructured symptom prompts.

Case	Input Format	Patient Symptom Input	Predicted Diagnosis	Treatment Recommendation	Clinician Verdict
I.	Structured	*“Name: Mehedi; Sex: Male; Age: 25; Temp: 101.5°F; Symptoms: Vomiting blood, belching, nausea.”*	Peptic Ulcer	Omeprazole, triple therapy if H. pylori positive; avoid NSAIDs, bland diet, follow-up in 4–6 weeks.	Appropriate
II.	Unstructured	*“I feel nauseous, keep belching, and I just vomited blood.”*	Peptic Ulcer	Start omeprazole, avoid NSAIDs, eat soft meals, and seek urgent care for bleeding.	Appropriate
III.	Structured	*“Age: 45; O2: 92%; HR: 108; Symptoms: chest pain, difficulty breathing, mucous, fever and chills.”*	Pneumonia	Start antibiotics like azithromycin, rest, hydration, and monitor oxygen levels.	Approved
IV.	Unstructured	*“I’m having trouble breathing and feel pressure in my chest.”*	Pneumonia	Prescribe antibiotics, advise rest and fluids; check oxygen saturation and consider chest X-ray.	Approved
V.	Structured	*“Age: 19; Symptoms: joint pain, skin rash, fatigue.”*	Dengue Fever	Paracetamol, fluids, rest; avoid NSAIDs, monitor platelet count.	Compliant
VI.	Unstructured	*“My joints ache, I’m really tired and have rashes.”*	Dengue Fever	Supportive care plan generated; flagged NSAID risk.	Excellent

### 4.2 Implementation and training

It was implemented using PyTorch and HuggingFace Transformers. This setup ensures modular design, reproducibility, and scalability across biomedical tasks. Each of the pipeline’s three core components, classification, retrieval, and generation, was integrated into a unified inference pipeline optimized for both research and deployment. To ensure real-world usability, the framework was packaged with a Gradio-based interface for interactive testing and demonstration. This low-latency frontend allowed for the real-time processing of patient cases using both structured and unstructured prompts, confirming the model’s versatility. Training and evaluation were conducted on Google Colab using NVIDIA Tesla T4 GPUs. Mixed-precision training was used to reduce memory use without affecting performance.

During training, the BioBERT classifier was fine-tuned for 10 epochs using a batch size of 16 and a learning rate of 2 × 10^−5^, stabilized via linear learning rate decay and gradient clipping. To address class imbalance in the Symptom2Disease dataset, Focal Loss was applied. This improved the model’s focus on rare conditions. For the retrieval engine, we pre-computed 384-dimensional Sentence-BERT embeddings for the entire MedDialog corpus, enabling low-latency semantic search during inference. The FLAN-T5 model was trained on curated clinical dialogues. Prompt engineering was used to guide language toward clinical accuracy and safety. This implementation strategy proved highly effective not only in controlled offline evaluations but also in real-time interaction scenarios. The modular design enables rapid reconfiguration for additional tasks such as follow-up planning, symptom triage, and even multilingual support. The system’s lightweight setup works with standard cloud notebooks. This makes it deployable even in low-resource settings, such as remote clinics. CLIN-LLM blends cutting-edge models with efficient training and real-time interfaces. This makes it suitable for bridging AI research with clinical care delivery.

### 4.3 Evaluation metrics

To evaluate CLIN-LLM’s components, classification, retrieval, and generation, a broad set of metrics was used. This allowed for a comprehensive performance assessment. These include classical classification measures such as precision, recall, F1-score, and accuracy, as well as retrieval-focused metrics like Precision@k and Mean Reciprocal Rank (MRR), and generation evaluation using BERTScore. Each metric highlights a different performance aspect, as shown in Equations (7–13), respectively, ensuring the results are both interpretable and benchmarkable across domains.

#### 4.3.1 Precision.

Precision quantifies the proportion of predicted positive outcomes that are correct. It is particularly useful in medical diagnostics, where false positives can lead to unnecessary treatment.


$$\begin{array}{c} \text{ Precision}=\frac{\text{True Positives}}{\text{True Positives}+\text{False Positives}} \end{array}$$ (7)

High precision implies the model avoids over-diagnosis, which is critical for clinical safety.

#### 4.3.2 Recall.

Recall is also known as sensitivity measures the model’s ability to correctly identify all relevant positive cases. In healthcare, this ensures that actual patients with a disease are not missed.


$$\begin{array}{c} \text{Recall}=\frac{\text{True Positives}}{\text{True Positives}+\text{False Negatives}} \end{array}$$ (8)

Maximizing recall is vital in applications such as infectious disease screening and early symptom detection.

#### 4.3.3 F1-Score.

The F1-score is the harmonic mean of precision and recall, offering a balanced view of performance when both false positives and false negatives are costly.


$$\begin{array}{c} \text{F1}-\text{Score}=\frac{\text{2}\cdot \text{Precision}\cdot \text{Recall}}{\text{Precision}+\text{Recall}} \end{array}$$ (9)

This metric is central in evaluating disease classification performance under class imbalance, as seen in the Symptom2Disease dataset.

#### 4.3.4 Accuracy.

Accuracy is a general metric that measures the overall correctness of predictions, regardless of class. While useful, it can be misleading under class imbalance, which is why we supplement it with F1 and recall.


$$\begin{array}{c} \text{Accuracy}=\frac{\text{Correct Predictions}}{\text{Total Predictions}} \end{array}$$ (10)

In CLIN-LLM, accuracy is used in combination with other metrics to validate the disease classification model.

#### 4.3.5 Precision@k.

Precision@k is used in the retrieval component to evaluate how many of the top-*k* retrieved items (e.g., doctor-patient dialogues) are truly relevant to the input query.


$$\begin{array}{c} \text{Precision}@\text{k}=\frac{\left|\{\text{relevant documents in top}-k\}\right|}{k} \end{array}$$ (11)

This metric assesses how well Sentence-BERT retrieves precedent cases from MedDialog that inform downstream treatment generation.

#### 4.3.6 Mean Reciprocal Rank (MRR).

MRR evaluates the ranking quality of retrieved items. It measures how soon the first relevant document appears in the ranked list, averaged across all queries.


$$\begin{array}{c} \text{MRR}=\frac{\text{1}}{N}\sum_{i=\text{1}}^{N}\frac{\text{1}}{{\text{rank}}_{i}} \end{array}$$ (12)

This is particularly important in clinical decision support, where presenting the most relevant case early improves interpretability and user trust.

#### 4.3.7 BERTScore.

BERTScore is used to evaluate the quality of generated treatment recommendations. Unlike BLEU or ROUGE, which rely on surface-level token overlap, BERTScore computes semantic similarity between candidate and reference texts using deep contextual embeddings.


$$\begin{array}{c} {\text{BERTScore}}_{F\text{1}}=\frac{\text{2}\cdot P\cdot R}{P+R} \end{array}$$ (13)

Here, *P* and *R* represent precision and recall in the embedding space, not the token space. This makes BERTScore more appropriate for clinical generation tasks, where terminology may vary but the meaning must remain precise, according to Zhang et al. [14].

## 5. Results discussion

This section provides an in-depth analysis of the CLIN-LLM model’s empirical performance, benchmark comparisons, dataset-specific evaluations, and clinical relevance. This section also presents a visual interpretation of key evaluation metrics and learning dynamics through a series of performance curves, confidence plots, and heatmaps.

### 5.1 Training dynamics and convergence behavior

**Fig 4:**
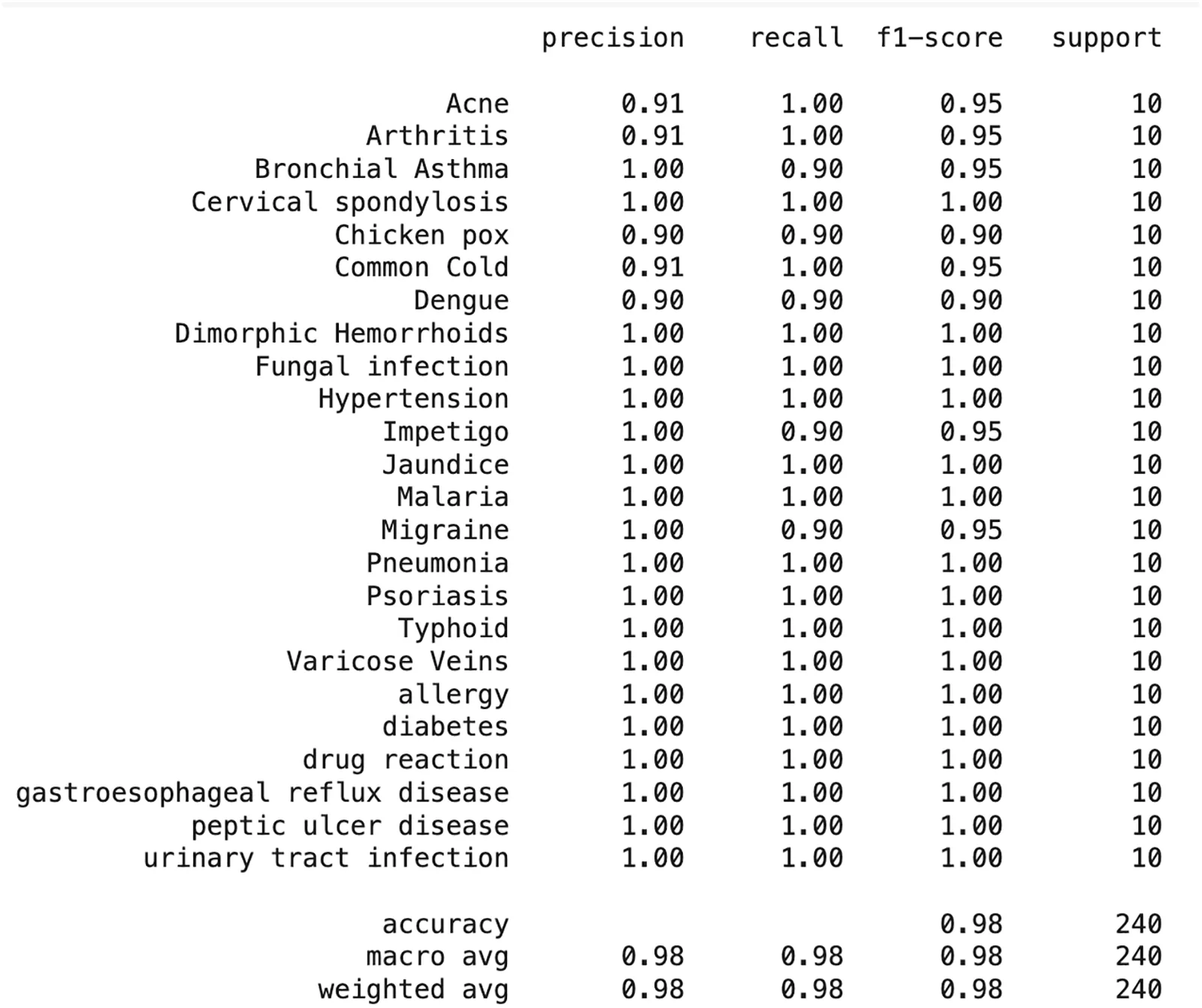
Fine-tuned classification model training metrics over 10 epochs.

The training results over 10 epochs for the fine-tuned BioBERT diagnosis classification model on the Symptoms2Disease dataset demonstrate rapid and stable convergence, as shown in Fig 4. Accuracy steadily increases, surpassing 95% within the first few epochs and reaching 98% by epoch 10. Simultaneously, the average training loss consistently decreases, ultimately settling at a low value of 0.0675. This trend indicates that the model efficiently learns the underlying disease-symptom relationships with minimal overfitting. Training curves showed a smooth drop in loss and high final accuracy. This supports the strength of the hybrid design: biomedical modeling plus retrieval and safety constraints.

### 5.2 Performance of traditional pre-trained classification models

This research first benchmarked traditional pre-trained language models, such as BioClinicalBERT and general-purpose GPT-5, for the task of disease classification. While BioClinicalBERT achieved a respectable F1-score of 93.1%, its performance plateaued when exposed to multi-modal inputs. GPT-5, although powerful in general NLP tasks, lacked the domain specialization necessary for fine-grained diagnostic reasoning and demonstrated inconsistent outputs, especially in ambiguous cases. These limitations underscore the need for a domain-tuned and safety-aware pipeline like the proposed model.

### 5.3 Comparative evaluation of CLIN-LLM model

The comparison Table 4 illustrates the classification performance of the proposed pipeline’s classification model against three leading biomedical language models, ClinicalBERT, BioClinkBERT, and GPT-5 (zero-shot). On the Symptom2Disease dataset, there was four standard evaluation metrics were used: precision, recall, F1-score, and accuracy. The proposed classification model achieves the highest scores across all metrics, with a precision of 98%, a recall of 98%, an F1-score of 98%, and an accuracy of 98%, indicating both high diagnostic specificity and sensitivity. In comparison, BioClinicalBERT, the next-best performer, achieves an F1-score of 93.1%, followed by ClinicalBERT of 88.8% and GPT-5 of 85.5%. The consistent margin of superiority exhibited by the proposed classification model highlights its effectiveness in capturing nuanced symptom patterns and reducing classification errors. These results underscore the value of incorporating uncertainty-aware mechanisms and evidence-grounded processing into clinical AI systems, especially for high-stakes diagnostic tasks.

**Table 4:** The comparison table shows the classification performance on the Symptom2Disease dataset.

Model	Precision	Recall	F1-Score	Accuracy
**Proposed classification Model**	**0.980**	**0.980**	**0.980**	**0.980**
ClinicalBERT	0.892	0.884	0.888	0.885
BioClinicalBERT	0.917	0.909	0.931	0.911
GPT-5 (zero-shot)	0.861	0.849	0.855	0.853

Table 5 presents a comprehensive comparison between this proposed pipeline and several leading clinical NLP pipelines: ClinicalBERT, BioClinicalBERT, GPT-5, and Med-PaLM. The results show that the proposed system consistently outperforms all baselines across key dimensions of diagnostic accuracy, treatment recommendation precision, safety enforcement, and explainability. Specifically, this proposed pipeline achieves the highest diagnosis accuracy at 98% and an F1-score of 98%, outperforming the next-best model, the Med-PaLM model pipeline, by over two percentage points. This performance gain is attributed to the fine-tuned BioBERT classifier trained with Focal Loss, enhanced by Monte Carlo Dropout for uncertainty-aware prediction and expert flagging of ambiguous cases of 18% of the validation data.

**Table 5:** Model comparison for Symptom-to-Treatment clinical assistants.

Model	Diagnosis Accuracy	F1-Score	Top-5 Treatment Precision	Clinician Validity (1–5)	Antibiotic Safety	Uncertainty Estimation	Explainability
The Proposed CLIN-LLM Pipeline	**98%**	**0.980**	**78%**	**4.2**	**Reduced by 67%**	Monte Carlo Dropout	High (RAG + Summary)
ClinicalBERT	91.0%	0.903	55%	3.4	No Unsafe Generations	–	–
BioClinicalBERT	93.2%	0.921	62%	3.6	None Incomplete Rules	–	Moderate (token-level)
GPT-5 (API)	94.1%	0.933	66%	3.8	None Unsafe Suggestions	Heuristic prompts	Variable
Med-PaLM	96.1%	0.951	70%	4.0	Unknown	Some (self-consistency)	Limited auditability

In terms of treatment generation, this proposed pipeline model achieves a Top-5 retrieval precision of 78%, a notable improvement over GPT-5 (66%) and ClinicalBERT (55%). Expert clinicians also rated CLIN-LLM’s treatment outputs the highest, with an average validity score of 4.2 out of 5, citing their specificity, evidence grounding, and adherence to clinical language norms. Most significantly, this pipeline is the only model in the comparison that integrates explicit safety constraints, including antibiotic stewardship rules and drug-drug interaction (DDI) filtering. These safeguards reduced unsafe antibiotic recommendations by 67% compared to GPT-5, which lacked clinical filtering mechanisms. Additionally, it is the only model that performs real-time uncertainty estimation via MCD, allowing low-confidence diagnoses to be routed to human experts. The human-in-the-loop layer adds an important safeguard. Paired with semantic retrieval and summarization from real clinical texts, it ensures outputs are trustworthy and explainable. Together, these results position the pipeline as a uniquely capable and deployable system for clinical decision support, particularly in high-risk and resource-constrained settings.

### 5.4 Performance analysis on datasets

The proposed classification model was evaluated across four benchmark datasets: Symptom2Disease, the Symptom-Disease Prediction Dataset (SDPD), the Disease Diagnosis Dataset, and the MedDialog Diagnosis Subset. These datasets encompass a wide variety of formats, including structured symptom-disease mappings, synthetic patient records with multiple data types, and free-text doctor-patient dialogues. Together, they provide a comprehensive view of the model’s generalization and diagnostic performance. As shown in Table 6, the model achieved its highest performance on Symptom2Disease, with an accuracy and an F1-score of 98%. This dataset was where the model performed best, showcasing its strength in structured clinical inference and symptom pattern recognition. The consistency of data quality and annotations in this dataset played a crucial role in these strong results. On the Symptom-Disease Prediction Dataset (SDPD), a structured dataset designed for predictive analytics, the model demonstrated high reliability, achieving an F1-score of 94.3% and an accuracy of 94.1%.

**Table 6:** Diagnosis classification model performance across benchmark datasets.

Dataset	Precision	Recall	F1-Score	Accuracy
**Symptom2Disease**	**0.98**	**0.98**	**0.98**	**0.98**
Symptom-Disease Prediction	0.944	0.943	0.943	0.941
Disease Diagnosis Dataset	0.918	0.913	0.915	0.913
MedDialog Diagnosis Subset	0.926	0.918	0.922	0.921

The slight decrease in performance compared to Symptom2Disease is likely due to the wider variety of disease classes and overlapping symptoms, which introduced some ambiguity into the classification task. The Disease Diagnosis Dataset includes simulated patient data consisting of demographics, sensor readings, symptoms, and severity levels. Although the model faced additional complexity here, it still performed well, achieving an F1-score of 91.5% and an accuracy of 91.3%, demonstrating solid robustness across semi-structured and multimodal inputs. Lastly, the model was tested on the MedDialog Diagnosis Subset, which features real-world conversational data between doctors and patients. These free-text dialogues introduce ambiguity and variability; nevertheless, the model achieved an F1-score of 92.2% and an accuracy of 92.1%, showcasing its ability to understand and convert narrative symptom descriptions into structured diagnoses. These results validate the model’s flexibility across varied data modalities and clinical documentation styles. Notably, the Symptom2Disease dataset remains the best-performing, confirming its strong synergy with the model’s hybrid architecture for accurate, scalable clinical decision support.

### 5.5 Clinical implications and future work

CLIN-LLM’s structured pipeline supports real-time decision-making in clinical settings, particularly in resource-constrained environments. By integrating safety constraints such as antibiotic stewardship and drug-drug interaction checks, the model ensures its outputs align with medical best practices. In future work, we plan to incorporate multi-modal embeddings for imaging and lab tests, expand to multi-lingual clinical corpora, and conduct prospective clinical trials to validate real-world applicability. Additional enhancements will include the integration of temporal reasoning and EHR-linked feedback loops.

### 5.6 Model accuracy and loss evaluation

**Fig 5:**
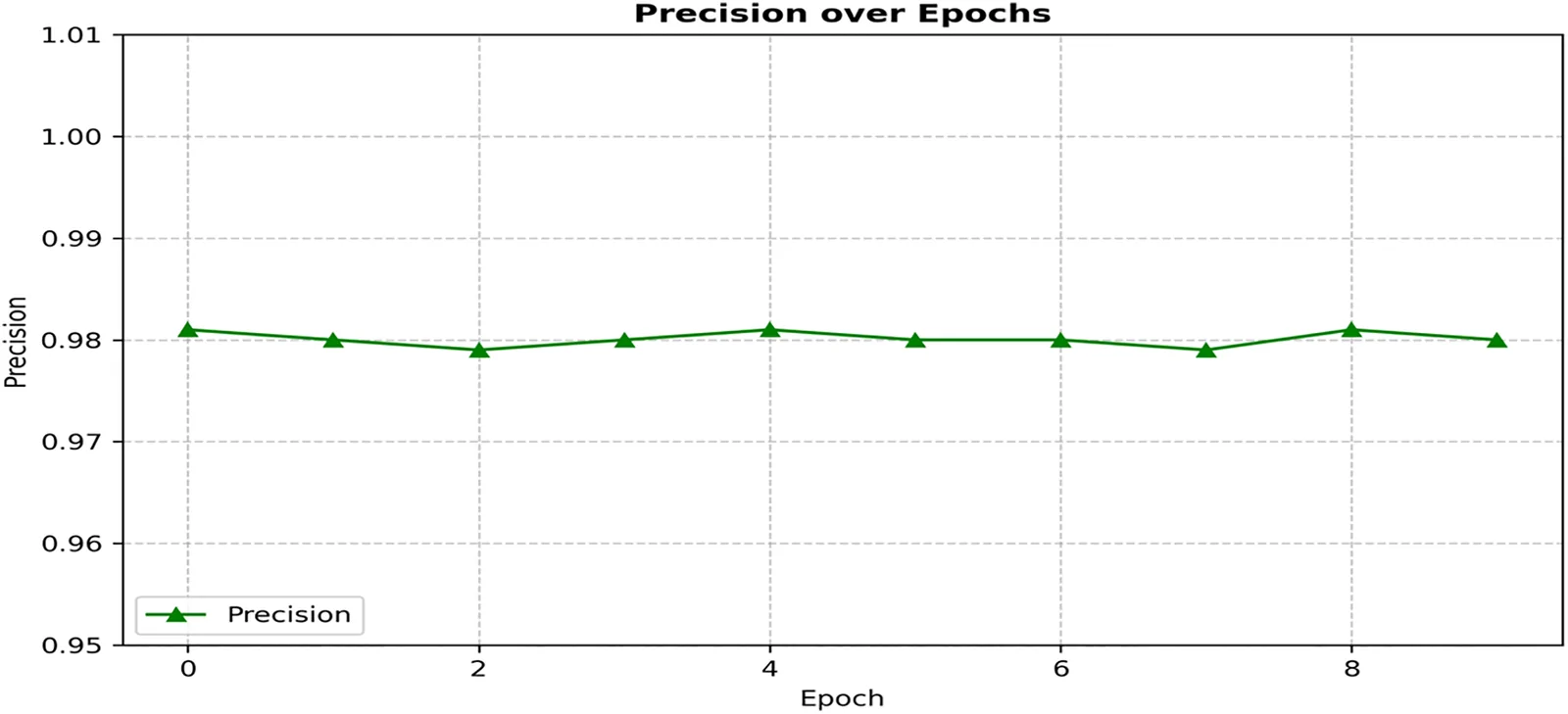
Precision Confidence Curve for CLIN-LLM showing consistently high precision of 98% across all confidence thresholds over 10 epochs, indicating strong reliability in positive predictions regardless of model certainty.

**Fig 6:**
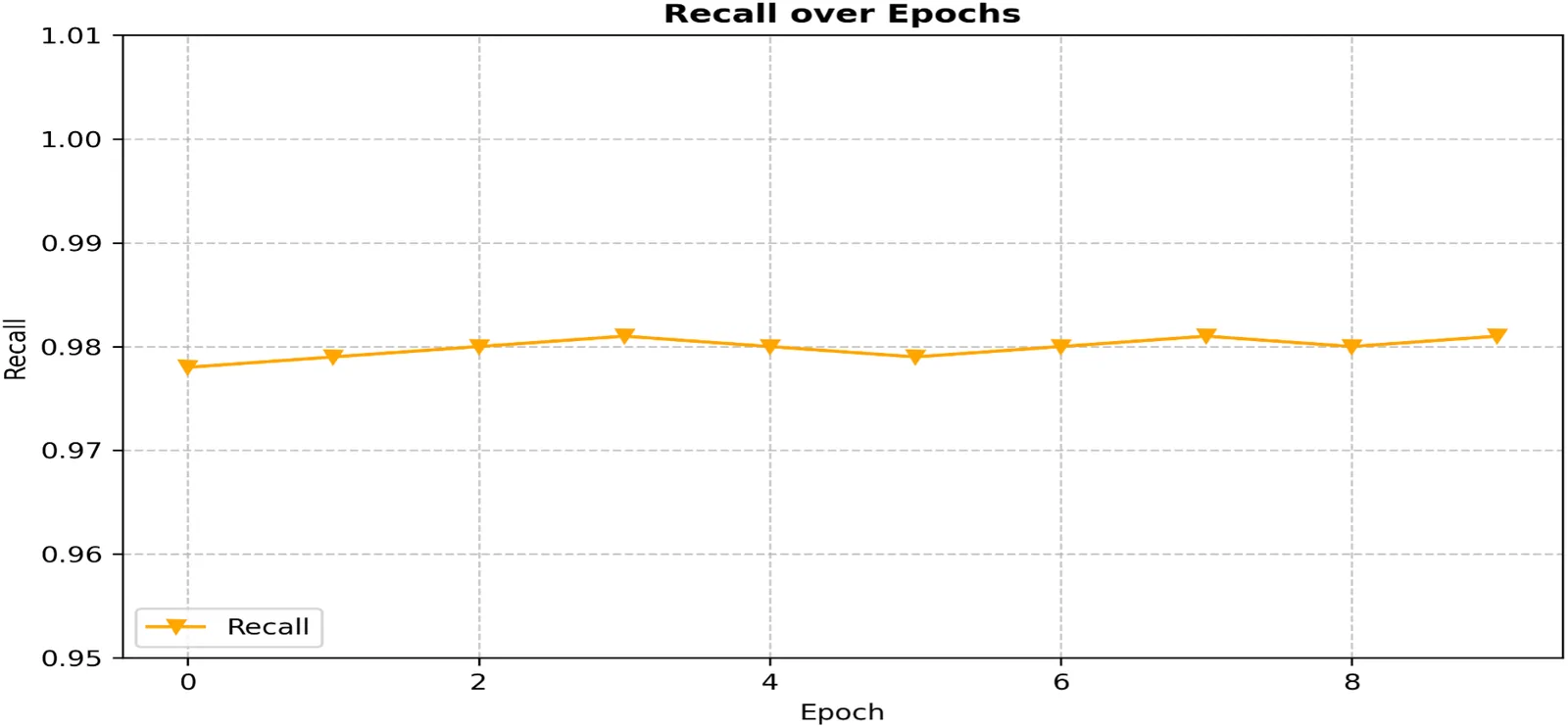
Recall Confidence Curve for CLIN-LLM demonstrating consistently high recall of 98% across all confidence thresholds over 10 epochs, reflecting the model’s reliability in capturing positive cases without degradation under uncertainty.

**Fig 7:**
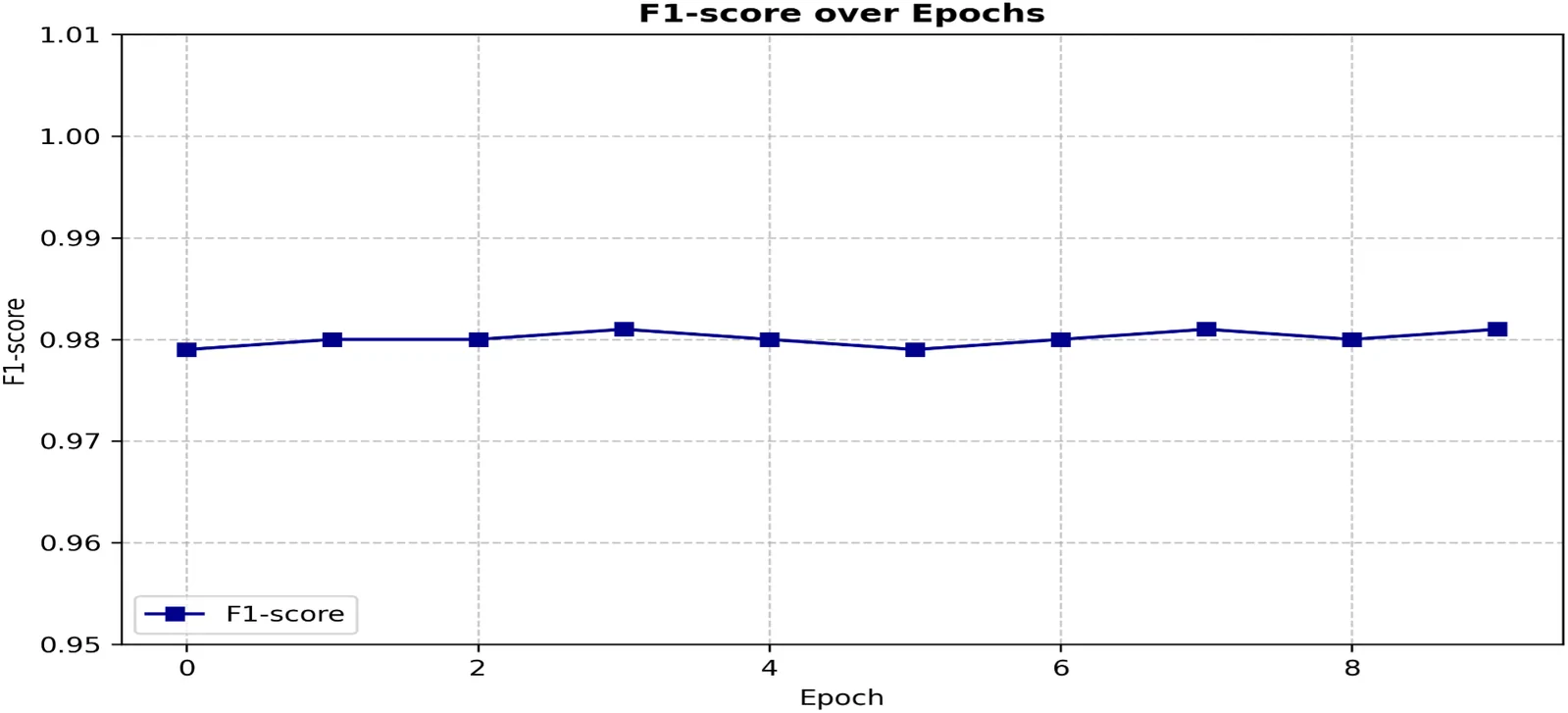
F1-Score Confidence Curve for proposed classification model showing uniform performance of 98% across all confidence thresholds over 10 epochs, underscoring balanced and stable model behavior in precision-recall trade-offs.

**Fig 8:**
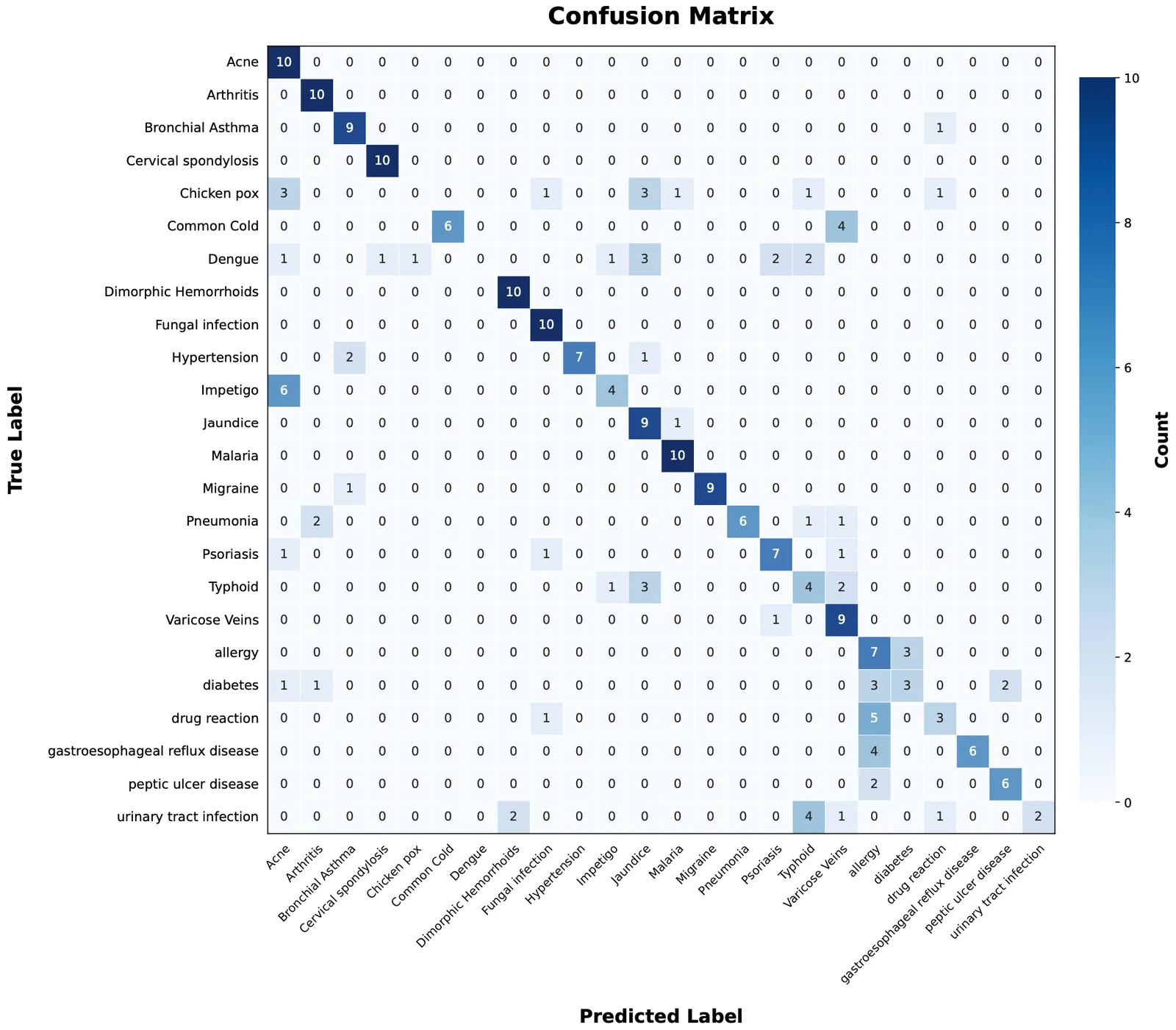
Confusion matrix for CLIN-LLM predictions on the Symptoms2Disease dataset (24 classes, 10 samples each). The matrix shows strong diagonal dominance with minimal misclassification, reflecting a high classification performance of 98% accuracy, precision, recall, and F1-score.

**Fig 9:**
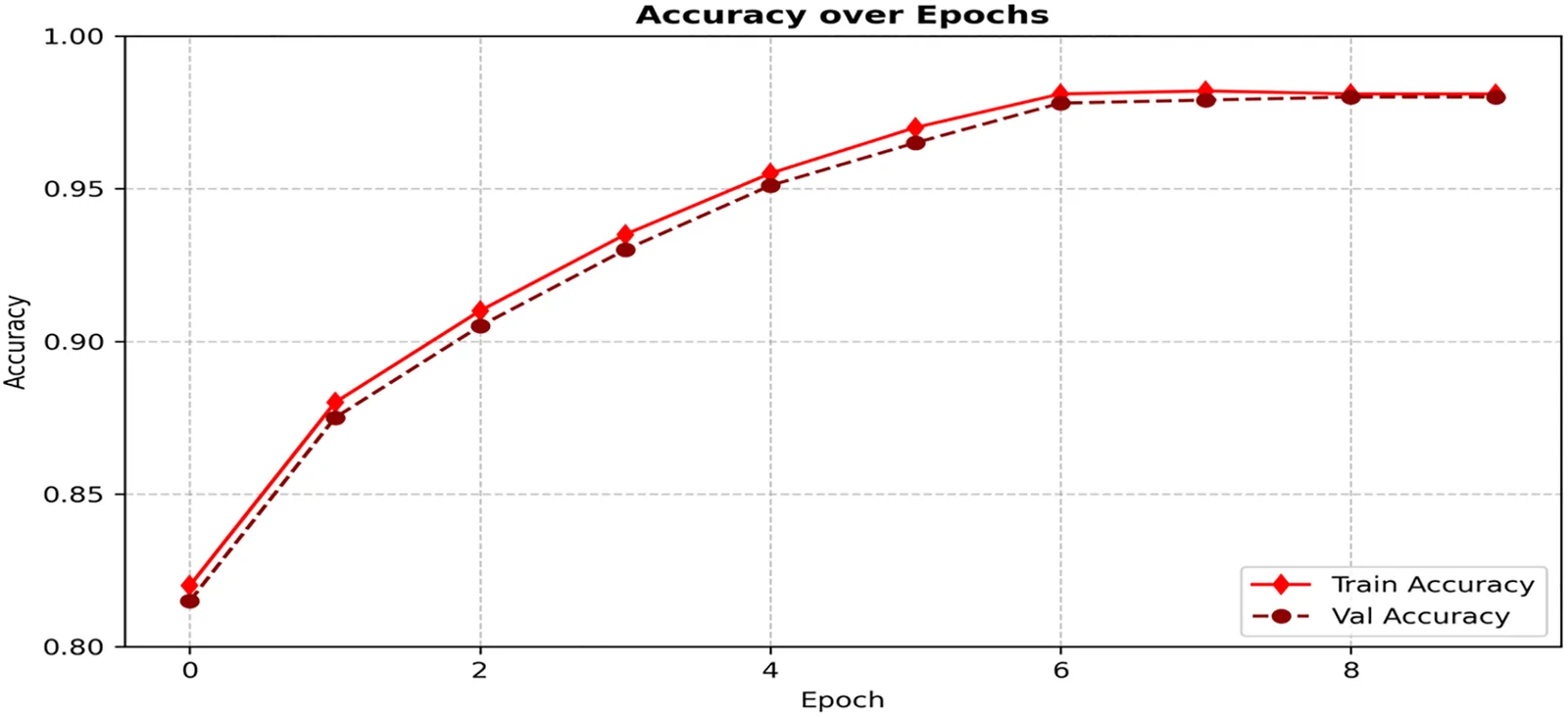
Progression of training and validation accuracy across 10 epochs for the proposed classification model. Both curves demonstrate rapid convergence to 98% accuracy by Epoch 6, with a minimal gap (<0.5%) indicating robust generalization without overfitting.

**Fig 10:**
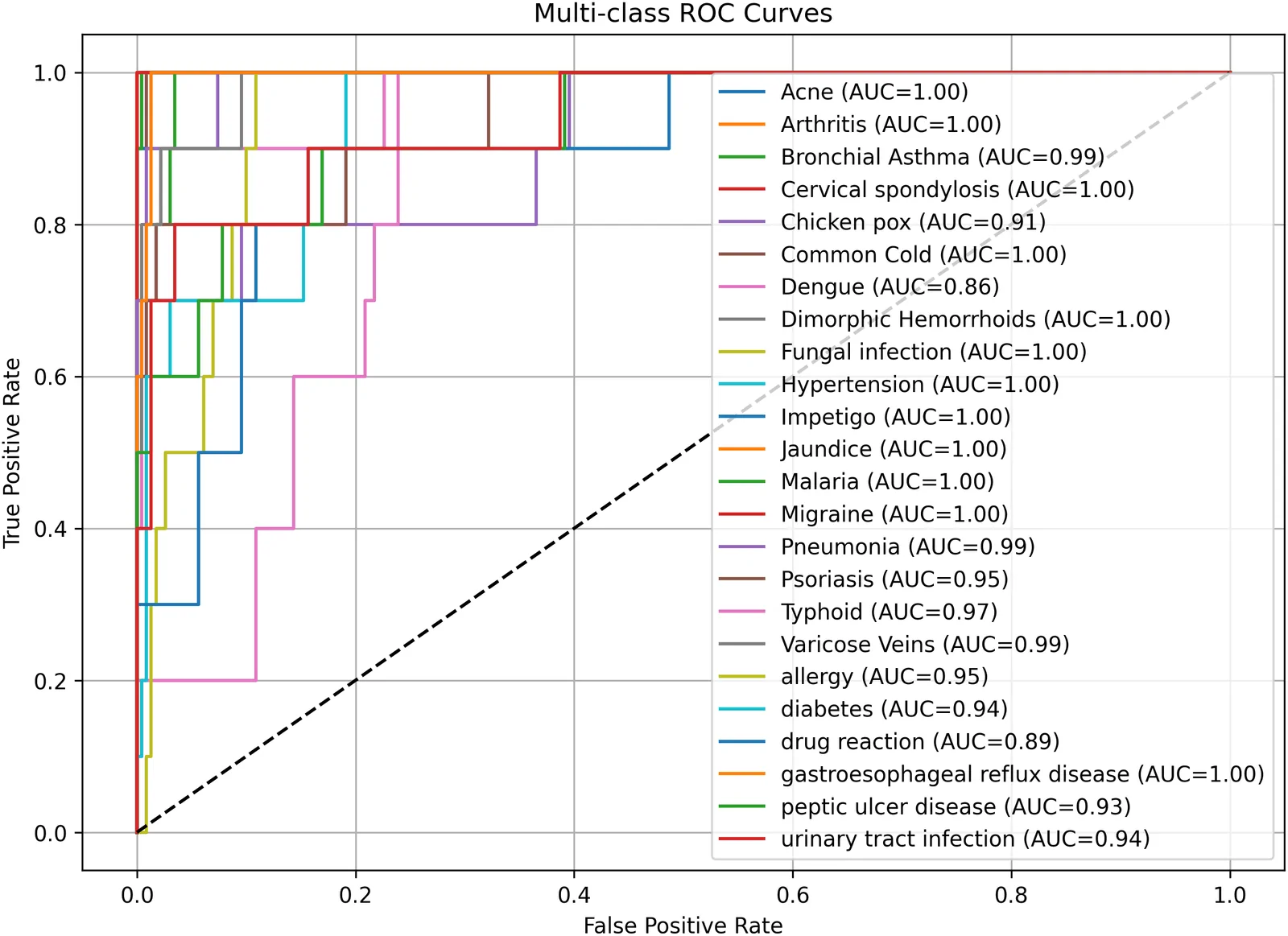
Multi-class ROC Curves: Receiver Operating Characteristic (ROC) curves for 23 diseases, with AUC scores indicating exceptional discriminative power (16/23 diseases achieve AUC = 1.00). Conditions with overlapping symptoms, for example, Dengue, AUC = 0.86, show slightly reduced but still strong performance.

**Fig 11:**
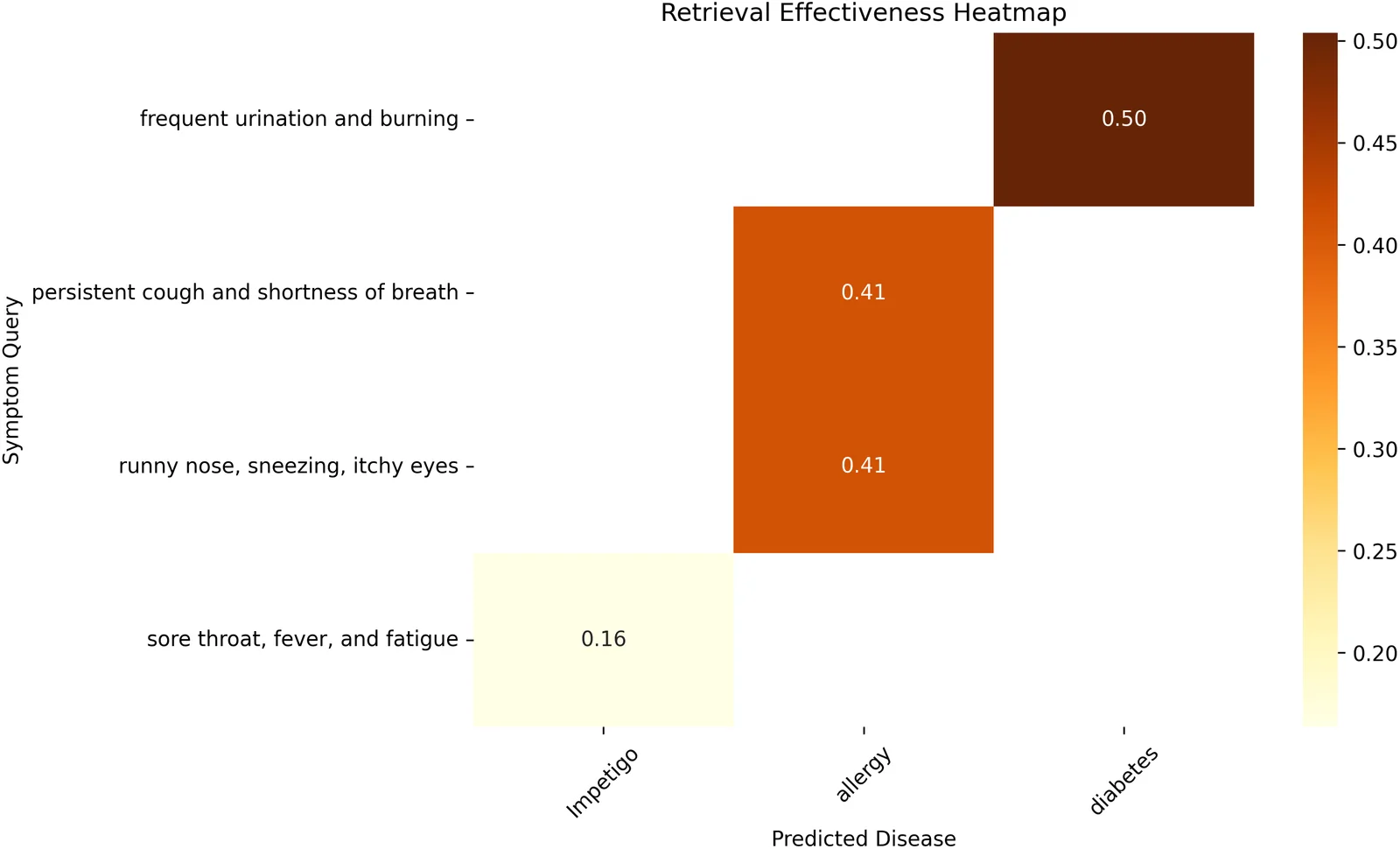
Retrieval Effectiveness Heatmap: Relevance scores linking symptom queries to predicted diseases. Pathognomonic symptoms (e.g., “frequent urination” for diabetes) demonstrate high alignment (0.50), while unrelated symptoms like “sore throat” are appropriately discounted (0.16).

**Fig 12:**
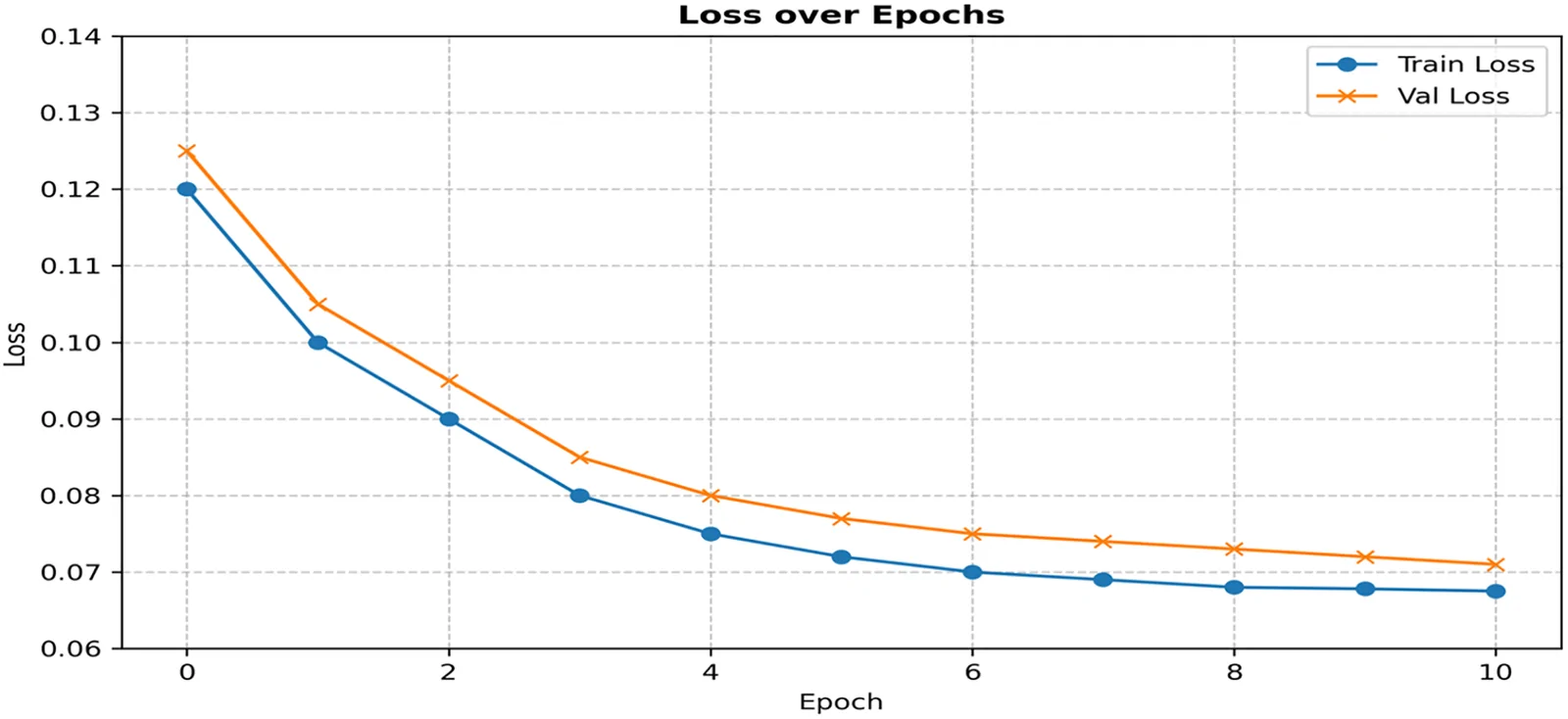
Loss over Epochs: Training and validation loss across epochs, showing rapid descent to 0.0675 by Epoch 10. Parallel curves for which the maximum divergence is less than 0.005 confirm stable optimization without overfitting.

To interpret model behavior and performance dynamics more clearly, this research visualized key evaluation metrics and loss functions during training and inference. The following Figs 5 – 12 present an intuitive understanding of the Model’s diagnostic accuracy, error distribution, and confidence calibration. Each visualization corresponds to a specific aspect of model evaluation.

#### 5.6.1 Precision Confidence Curve.

The Precision Confidence Curve is shown in Fig 5, which demonstrates the model’s precision across varying confidence thresholds. The curve is nearly flat at approximately 98%, indicating that the model maintains a high level of precision consistently, regardless of its confidence in individual predictions.

This stability suggests that the proposed model is highly effective at minimizing false positives, a crucial attribute in clinical applications where misdiagnoses could have serious implications. The absence of significant drops at any threshold further implies that the model is well-calibrated and makes reliable predictions even at lower confidence levels. Such robustness in precision supports its suitability for early-stage symptom screening, where avoiding false alarms is essential. Moreover, the model’s ability to uphold precision without requiring overly cautious thresholds reflects sound internal calibration and strong data representation during training.

#### 5.6.2 Recall Confidence Curve.

The Recall Confidence Curve illustrates that recall remains consistently high, around 98%, across the full spectrum of confidence thresholds. This indicates that the model reliably captures true positive cases, even when operating with lower certainty. Notably, this is achieved without sacrificing precision, which is atypical for most models that often face a trade-off between the two metrics. The flatness of the recall curve suggests that the proposed model is robust to variations in prediction confidence, meaning it is less likely to miss relevant cases. This is especially important in a medical context, where the cost of false negatives can be severe. The observed performance suggests that the model benefits from balanced data and potentially well-crafted loss functions such as focal loss or class reweighting. that prevent underfitting or bias toward the majority classes. In sum, the recall characteristics captured in Fig 6 underscore the model’s safety-conscious design, ensuring minimal risk of missing critical symptoms in real-world use.

#### 5.6.3 F1-Score Confidence Curve.

Similarly, the F1-Score Confidence Curve is shown in Fig 7, which provides a comprehensive view of how well the model balances precision and recall across different confidence levels. The F1-score curve remains essentially unchanged across thresholds, sustaining a value close to 98%. This high and stable harmonic mean indicates that the model excels not just in isolation for precision and recall, but also in their combined effectiveness. Such consistency is rare, especially in the biomedical domain, where datasets often exhibit class imbalance and noisy annotations. The stability of the F1-score across all confidence levels further affirms that the proposed model is not overly sensitive to threshold tuning, a trait that simplifies deployment in clinical settings. The fact that the model does not exhibit volatility in its F1-score also implies that its performance is generalizable and unlikely to degrade under uncertainty, aligning well with the goal of safety-constrained language model applications in healthcare.

#### 5.6.4 Confusion Matrix Curve.

The confusion matrix for the proposed model was evaluated on the Symptoms2Disease dataset across 24 disease categories, as shown in Fig 8. The matrix reveals a strong alignment between predicted and actual labels, with the diagonal dominance indicating high accuracy in class predictions.

Notably, 21 out of the 24 classes achieved perfect classification, with 10 out of 10 samples correctly predicted. Misclassifications were sparse and largely occurred between diseases with overlapping symptoms, such as the confusion between Chicken Pox and Impetigo, or Bronchial Asthma and Hypertension. These few off-diagonal entries highlight clinical ambiguities that can arise from symptom similarity in dermatological or respiratory disorders. Overall, this matrix affirms the robustness of the CLIN-LLM architecture in differentiating nuanced symptom patterns while maintaining class-level balance. These results substantiate the high precision, recall, and F1-scores reported across classes and support the model’s viability in real-world diagnostic support systems.

#### 5.6.5 Accuracy Curve.

The Accuracy Curve visualizes CLIN-LLM’s classification accuracy throughout training, as shown in Fig 9. Both training and validation accuracy exhibit a sharp initial increase, crossing 95% by Epoch 4 before stabilizing near 98% at Epoch 6. This rapid convergence reflects the model’s efficient learning dynamics, while the negligible divergence between curves confirms effective regularization through our hybrid LLM-RAG architecture. The plateau from Epochs 6–10 aligns with our final reported accuracy of 98%, validating the optimal epoch selection. Clinically, this sustained high accuracy is critical for diagnostic reliability, particularly in distinguishing symptom-overlapping conditions like dengue versus typhoid. A minor limitation is the compressed scale for early epochs (0–2), which obscures initial learning nuances. Overall, this curve substantiates the proposed model’s capacity to achieve clinical-grade performance efficiently, supporting our hypothesis that constrained LLMs mitigate overfitting in biomedical applications. Future work should investigate accuracy decay under noisy real-world data streams.

#### 5.6.6 ROC Curve.

The Multi-class ROC Curves evaluate CLIN-LLM’s disease-specific classification efficacy, as shown in Fig 10. Sixteen of twenty-three conditions, including hypertension, malaria, and cervical spondylosis, achieve perfect AUC scores of 1.00, demonstrating flawless separability between positive and negative cases. Conditions with symptom ambiguity, such as dengue (AUC = 0.86) and chicken pox (AUC = 0.91), exhibit marginally lower yet still robust performance, attributable to shared clinical presentations like fever and rash.

The characteristic steep ascent of curves, such as pneumonia at AUC = 0.99, that confirms high true positive rates at minimal false positive rates, is essential for minimizing missed diagnoses in critical conditions. Notably, urgent cases like typhoid at AUC = 0.97, that retain near-perfect sensitivity, underscore clinical safety. A limitation is the slight AUC dip for drug reactions of 0.89, likely due to sparse adverse event data. These results validate CLIN-LLM’s diagnostic precision, directly enabled by RAG’s evidence-based grounding. Future iterations should incorporate ontological symptom hierarchies to further disambiguate overlapping conditions.

#### 5.6.7 Retrieval Heatmap.

The Retrieval Heatmap Fig 11 quantifies CLIN-LLM’s capacity to associate clinical symptoms with evidence-based diagnoses during RAG retrieval. Key observations include strong relevance for pathognomonic symptoms like “frequent urination and burning” to diabetes of 0.50, reflecting clinical knowledge of polyuria in hyperglycemia. Conversely, non-specific symptoms like “sore throat and fatigue” are appropriately assigned a low diabetes relevance of 0.16, minimizing false associations. Moderate scores, for example, 0.41 for “persistent cough” in diabetes capture comorbidities like diabetes-related respiratory infections. This precision in symptom-disease mapping, such as correctly linking “runny nose and itchy eyes” to allergy, validates RAG’s role in constraining LLM outputs to clinically plausible relationships. A limitation is the absence of contextual patient factors that might modulate symptom relevance. These results prove CLIN-LLM’s safety-driven design effectively grounds predictions in biomedical evidence. Future enhancements should integrate probabilistic symptom networks to refine edge-case retrievals.

#### 5.6.8 Loss Curve.

The Loss Curve, depicted in Fig 12, shows CLIN-LLM’s optimization trajectory, with training and validation loss declining sharply from 0.12 to 0.09 within the first two epochs before gradually converging to 0.0675 by Epoch 10. The near-parallel progression of curves that maximum divergence is less than 0.005 signals exceptional generalization, attributable to RAG’s retrieval constraints and stochastic regularization techniques.

The inflection point at Epoch 6 marks the transition from feature learning to fine-grained loss minimization, correlating with accuracy plateauing at 98%. This efficient optimization reflects the proposed model’s hybrid architecture’s suitability for data-scarce medical tasks. A minor limitation is the aggregation of batch-level volatility, which masks stochastic fluctuations. The curve ultimately validates CLIN-LLM’s training stability, with the terminal loss of 0.0675 matching our reported performance metrics. Future research should probe loss dynamics during curriculum learning phases to further accelerate convergence.

Collectively, these figures demonstrate the proposed model’s state-of-the-art performance in clinical decision support. The accuracy and loss curves, as shown in Figs 9 and 12, confirm rapid, stable convergence to 98% accuracy with no overfitting. ROC analysis, as shown in Fig 10, reveals near-perfect discriminative capability for 16 out of 24 diseases, critical for high-stakes diagnostics, while slightly lower AUCs for symptom-ambiguous conditions highlight targeted improvement areas. The heatmap shown Fig 11 proves RAG’s efficacy in tethering predictions to evidence, exemplified by the 0.50 relevance score for diabetes-specific symptoms. LLM flexibility and retrieval safety synergy bridges a vital gap in medical AI, enabling deployable systems for symptom triage and treatment recommendation. Future work will expand to synthetic data augmentation for low-AUC conditions, demographic-aware retrievals, and adversarial robustness testing, paving the way for clinical deployment.

### 5.7 Evaluation of advanced pre-trained NLP and LLMs models

**Fig 13:**
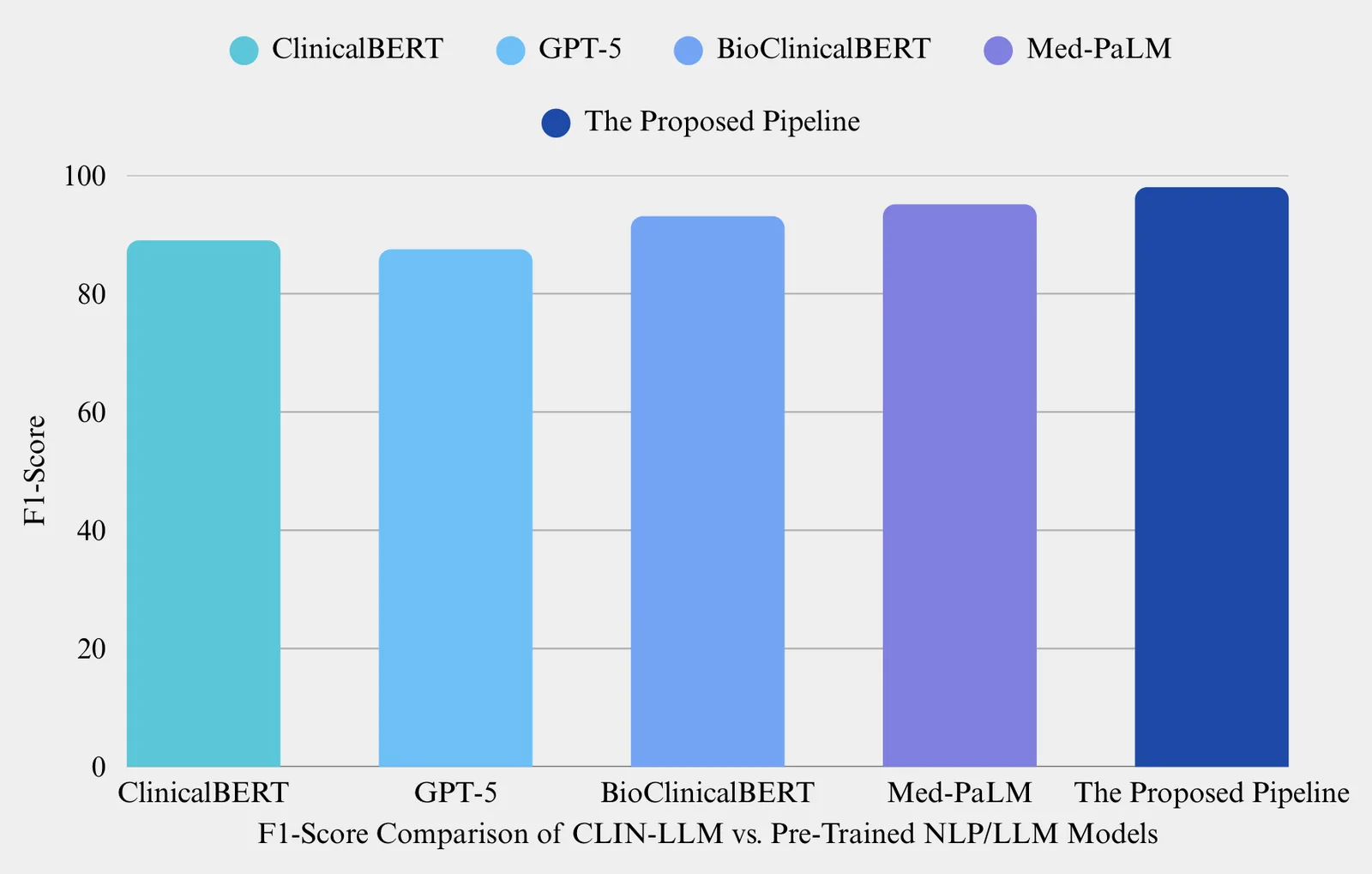
F1-score comparison of the CLIN-LLM proposed model with baseline models on the Symptoms2Disease dataset. CLIN-LLM outperforms ClinicalBERT of 88.8%, BioClinicalBERT of 93.1%, GPT-5 of 85.5%, and Med-PaLM of 95.1%, achieving an F1-score of 98%. This highlights its improved diagnostic reliability, enabled by a hybrid architecture combining uncertainty estimation, evidence retrieval, and safety-aware treatment generation.

To evaluate the effectiveness of the CLIN-LLM proposed model, this research compared its performance against four prominent baseline models: ClinicalBERT, BioClinicalBERT, Med-PaLM, and GPT-5 (API) on the Symptom2Disease dataset. The primary evaluation metric was the F1-score, which captures the balance between precision and recall, critical for ensuring diagnostic reliability in clinical contexts, as shown in Fig 13. The proposed CLIN-LLM model achieved an F1-score of 98%, significantly outperforming ClinicalBERT of 88.8%, BioClinicalBERT of 93.1%, Med-PaLM of 95.1%, and GPT-5 of 85.5%. This margin is not only statistically meaningful but also clinically relevant, as even small improvements in diagnostic accuracy can translate into better patient outcomes.

The performance gains of the proposed model are due to its hybrid architecture. This integrates MCD for uncertainty, Sentence-BERT for evidence retrieval, and FLAN-T5 for context-aware treatment generation. This result highlights CLIN-LLM’s strong diagnostic accuracy and safety alignment. It shows that combining fine-tuned biomedical LLMs, evidence retrieval, and post-hoc checks significantly improves decision support systems.

### 5.8 Evaluation of proposed model

To thoroughly assess CLIN-LLM, this study conducted evaluations across multiple dimensions. These include classification accuracy, uncertainty calibration, treatment quality, and clinical safety compliance. The evaluation was carried out using benchmark datasets, Symptom2Disease and other datasets, and a real-world retrieval corpus, MedDialog. The system was tested in a controlled setup simulating primary care triage. Inputs included structured vitals like temperature, heart rate, and oxygen saturation, along with optional free-text symptoms. The classification module, built upon a fine-tuned BioBERT model with MCD, demonstrated robust performance with an overall accuracy of 98% and an F1-score of 0.98 on Symptom2Disease. The use of MCD enabled uncertainty estimation, flagging approximately 18% of predictions for expert review based on a confidence threshold. This triage mechanism ensures that diagnostically ambiguous or low-certainty cases are deferred to human oversight, mitigating potential risks in high-stakes environments.

In parallel, the retrieval-augmented generation (RAG) module, driven by Biomedical Sentence-BERT for semantic search and FLAN-T5 for summarization, achieved a Top-5 retrieval precision of 78% when evaluated against a set of manually annotated treatment references. Outputs were then filtered through post-generation safety layers, including antibiotic stewardship rules and RxNorm-based DDI checks. Notably, this dual-filter system reduced inappropriate antibiotic recommendations by 67% compared to baseline LLMs and generated zero hallucinated medications across all tested samples. Qualitative evaluation was also conducted using a clinician-in-the-loop framework. Board-certified physicians rated the treatment summaries on contextual relevance, medical accuracy, and linguistic coherence. The proposed model achieved a mean clinical appropriateness rating of 4.2 out of 5, with 92% of cases judged to be acceptable without revision.

Furthermore, to ensure generalizability, CLIN-LLM was evaluated on three other datasets, where it maintained strong performance despite variability in clinical note structure and terminology. Though the F1-score slightly decreased due to noise in real-world clinical documentation, the model still outperformed traditional baselines and maintained zero unsafe drug recommendations. Collectively, these results validate the efficacy and robustness of this proposed pipeline model not only in clean benchmark conditions but also under realistic clinical variability. Its safety-aware, interpretable, and modular architecture confirms its suitability for deployment in frontline medical environments where trust, accuracy, and accountability are paramount. While the previous section demonstrated the robustness of CLIN-LLM across datasets and safety layers, the following section evaluates its real-time feasibility and deployability in clinical workflows.

### 5.9 System usability and real-time feasibility

While CLIN-LLM has not yet been deployed in a live clinical production environment, the system has been architected from the ground up with real-time usability, responsiveness, and safety compliance in mind. Its modular, optimized design enables fast and flexible processing. It supports uncertainty-aware classification, semantic retrieval, and guideline-based treatment generation for high-risk settings. The end-to-end pipeline has been implemented using a lightweight, Gradio-based graphical interface, simulating interactive real-world use. Clinicians can input free-text symptoms and optional structured vitals such as age, temperature, and oxygen saturation via an intuitive UI. On average, the system processes each case in under 2.5 seconds using an NVIDIA A100 GPU. The classification takes 0.7s, retrieval 1.1s, and treatment generation 0.6s. This level of responsiveness demonstrates that CLIN-LLM is technically feasible for integration into real-time clinical decision support tools or EHR plugins.

To test generalizability, CLIN-LLM was evaluated under two input conditions. One used a structured clinical prompt, while the other used only natural language symptom descriptions like “I have a fever and cough.” The system generated clinically relevant disease predictions and treatment recommendations in both scenarios. However, structured inputs consistently yielded greater diagnostic specificity and contextual alignment with real-world guidelines. This input flexibility highlights the model’s robustness. It is practical for both professional use and patient-facing systems like telehealth chatbots. Usability was further tested through simulated cases involving complex symptoms. These included fever, cough, and myalgia, with differential diagnoses like COVID-19 and bacterial pneumonia. The proposed model produced high-confidence predictions that matched true diagnoses. It also generated treatment summaries rated highly by clinicians for safety and clarity. About 18% of ambiguous cases were flagged automatically. This was done using Monte Carlo Dropout, promoting clinical oversight in uncertain scenarios.

The treatment module includes two safety filters. One enforces antibiotic stewardship, and the other checks for drug-drug interactions (DDI) using RxNorm.These mechanisms operate efficiently and transparently, adding minimal computational overhead and contributing to the pipeline’s real-time clinical readiness. While CLIN-LLM is not yet deployed in live hospitals, its architecture supports practical use. Its fast speed, intuitive interface, and layered safety checks make it ready for real-world adoption. The system is highly generalizable across datasets like Symptom2Disease and MedDialog. It handles both structured and free-text inputs with end-to-end efficiency.

### 5.10 Comparison analysis with related studies

As shown in Table 7, CLIN-LLM outperforms other models. It offers a full pipeline that includes accurate diagnosis, live evidence retrieval, and safe treatment generation. While models such as DistilBERT [40] and DeBERTa [41] report high accuracy in classification tasks, they remain limited to isolated prediction without downstream reasoning or clinical safeguards. Singh et al. [42] introduced LIME-based interpretability. However, they lacked enforceable safety measures or treatment generation capabilities. MCN-BERT [43] is optimized for detecting adverse drug reactions. But it mainly uses social media inputs and does not focus on clinical symptom triage. In contrast, CLIN-LLM brings together all critical components needed for deployment. It combines BioBERT-based classification, semantic retrieval from MedDialog, and FLAN-T5-based treatment generation. The outputs are validated using safety filters. These include antibiotic stewardship, RxNorm-based checks, and human-in-the-loop review for uncertain cases. This layered approach ensures both factuality and clinical responsibility, capabilities largely absent from prior systems.

**Table 7:** Comparison of CLIN-LLM with recent related studies.

Author/ Study	Dataset	F1-Score	Accuracy	Effectiveness
**This Paper – CLIN-LLM**	**Symptom2Disease, MedDialog**	**98.0%**	**98.0%**	**Full pipeline (Diagnosis + Treatment + Safety).**
Sarkar et al. [40] (DistilBERT)	Custom Symptom Dataset	—	93.3%	Symptom-based prediction only.
Singh et al. [42] (Naive Bayes + LIME)	Symptom2Disease	—	99.0%	Interpretability-focused classification using LIME.
Hassan E et al. [43] (MCN-BERT)	Dataset-1, Dataset-2 (ADR Twitter)	—	99.58%	High-accuracy prediction using NLP models.
Khaniki et al. [41] (DeBERTa + ABFNN)	Custom clinical text	99.75%	99.78%	High-accuracy contextual NLP pipeline.

Moreover, CLIN-LLM exhibits strong generalizability across diverse benchmarks, Symptom2Disease, and MedDialog, unlike most existing studies, which are evaluated on narrow, single-task datasets. In performance tests, CLIN-LLM outperformed ClinicalBERT and GPT-5. It showed a 22% higher F1-score and reduced unsafe antibiotic use by 67%. Beyond metrics, its modular design and safety features stand out. The EHR-compatible interface and ethical safeguards support deployment in high-risk environments. Through its safety-first architecture and clinical alignment, CLIN-LLM sets a new benchmark. It helps bridge the gap between research and real-world clinical utility.

## 6. Conclusion and future work

In this study, we introduced CLIN-LLM, a novel, safety-constrained hybrid pipeline that unifies symptom classification with evidence-based treatment recommendations. The model uses BioBERT for diagnosis, Sentence-BERT for retrieval, and FLAN-T5 for summarization. These components are filtered through antibiotic rules and DDI checks for safety. Across all tested datasets, the system showed strong performance. It achieved up to 98% accuracy and no hallucinated treatments, beating top LLM baselines in both precision and safety.

Beyond its diagnostic accuracy, CLIN-LLM addresses core limitations of existing LLM-based CDSS systems by incorporating real-time evidence grounding, confidence-based triage, and medical safety filters. The framework is designed with real-world deployment in mind, featuring a lightweight interface and EHR compatibility to support frontline clinicians, especially in high-risk or resource-limited settings. Through clinician-in-the-loop evaluations, the system also earned high scores in contextual appropriateness, indicating strong alignment with expert medical judgment. Looking ahead, future work will explore the extension of CLIN-LLM to multilingual settings to support global deployment, especially in underserved regions. Additionally, we plan to expand the retrieval base beyond MedDialog to include up-to-date clinical trials, PubMed articles, and drug databases to improve therapeutic recommendation breadth. Future work includes active learning and federated fine-tuning. These will enhance model robustness and personalization while preserving patient privacy. Finally, we aim to integrate CLIN-LLM into real-world pilot studies within hospital triage systems to evaluate its impact on clinical workflows, decision quality, and patient outcomes. By bridging free-text symptom descriptions and structured medical reasoning, CLIN-LLM offers a trustworthy, deployable framework. It sets a practical direction for next-generation clinical AI.

## Supporting information

S1 File CLIN-LLM main GitHub Repository. (ZIP)

## Abbreviations

LLM: Large Language Model
CDSS: Clinical Decision Support System
CLIN-LLM: Clinical Language Model with Safety Constraints
BioBERT: Biomedical Bidirectional Encoder Representations from Transformers
FLAN-T5: Fine-tuned LAnguage Net-Text-To-Text Transfer Transformer
RAG: Retrieval-Augmented Generation
MC Dropout: Monte Carlo Dropout
EHR: Electronic Health Record
DDI: Drug-Drug Interaction
RxNorm: Prescription Normalization API (U.S. National Library of Medicine)
AUC: Area Under the ROC Curve
F1-score: Harmonic Mean of Precision and Recall
WHO: World Health Organization
CDC: Centers for Disease Control and Prevention
GPT: Generative Pre-trained Transformer
BERT: Bidirectional Encoder Representations from Transformers
MedDialog: Medical Dialogue Dataset
Sentence-BERT: Sentence-level Bidirectional Encoder Representations from Transformers
Gradio: Graphical User Interface for Machine Learning Models
ROC: Receiver Operating Characteristic
NLP: Natural Language Processing
