# Supplementary material for: CLIN-LLM: A safety-constrained hybrid framework for clinical diagnosis and treatment generation
Source: PLoS One. 2026 Aug 27;21(8):e0348611. doi: 10.1371/journal.pone.0348611 (PMC13521392; doi:10.1371/journal.pone.0348611)
Supplement: Supplementary file 2 [file pone.0348611.s003.docx]

**Manuscript ID**: PONE-D-25-43435R1
**Title:** *CLIN-LLM: A Safety-Constrained Hybrid Framework for Clinical Diagnosis and Treatment Generation.*

**To:** Hui Li
**Academic Editor
PLOS ONE**

**Subject:** Point-by-Point Response to Editorial Comments

Dear Dr. Hui Li,

Thank you for allowing us to resubmit our manuscript after major revision. We sincerely appreciate the editor’s valuable feedback and have carefully addressed all the comments. Please find below our point-by-point responses. All modifications have been incorporated into the revised manuscript and are highlighted in yellow.

We look forward to your kind consideration of our revised manuscript.

Warm regards,
Rafid Mostafiz
(Corresponding Author)
On behalf of all co-authors

**Reviewer #1 – Point-by-Point Responses:**

**1.** The authors should address the concerns and limitations of the LLM-based model such as GPT. References such as *Frontiers in artificial Intelligence. 2023 Apr 5;6:1166014* should be included.

**Our Response:** We appreciate the reviewer's valuable suggestion. We have significantly expanded our critical analysis of LLMs in clinical contexts by introducing a dedicated section on safety and limitations. This discussion addresses "hallucinations," the lack of clinical common sense, and privacy risks. We have integrated the recommended reference to *Frontiers in Artificial Intelligence* [32] (cited as Wang et al. in the revised bibliography) to contextualize how these limitations necessitate our hybrid architecture.

**Location of Revision:** These changes are located in the Revised Manuscript under Section 5.5, " **Clinical Implications and Future Work**," in the paragraph. The revised text is highlighted in yellow in the marked-up manuscript.

**2. The authors should briefly review medical decision-making assisted by AI. Some references, such as *Algorithms. 2025 Mar 9;18(3):156* would be useful.**

**Our Response:** We appreciate this feedback. The Introduction and Literature Review have been updated to include a comprehensive trajectory of AI-assisted medical decision-making, from traditional rule-based systems to modern generative pipelines. By incorporating the suggested reference to *Algorithms* [37] (cited as Chen L. et al.), we highlight the current research gap between predictive accuracy and clinical interpretability.

**Location of Revision:** The revision can be found in the Revised Manuscript under Section 1, "Introduction," and Section 2, "Literature Review," specifically in the first paragraphs of these sections. All new text is highlighted in yellow.

**3. The current version of GPT is GPT-5. However, this study used GPT-3.5, which is out of date.**

**Our Response:** We have **fully redeveloped the CLIN-LLM framework using GPT-5**. Our revised results demonstrate that our hybrid pipeline achieves a 67% reduction in unsafe antibiotic suggestions compared to the standard GPT-5 model. This transition ensures our study reflects the most current state-of-the-art in generative AI.

**Location of Revision:** Updates regarding the GPT-5 integration are reflected throughout the Methodology, Results, and Abstract, all highlighted in yellow.

**4. Abbreviations should move to the end of the paper.**

**Our Response:** To improve narrative flow and adhere to standard technical reporting, we have removed the consolidated abbreviations list from the early sections. A comprehensive "List of Abbreviations" has been appended to the very end of the paper, immediately following the References.

**Location of Revision:** This change is located at the very end of the document in a new "Abbreviations" section following the Reference section. The list is highlighted in yellow in the marked-up manuscript.

**5. Monte Carlo simulation is based on random sampling, the authors should discuss why such a mathematical method can be used in medical decision making.**

**Our Response:** We have added a rigorous mathematical justification for employing Monte Carlo (MC) simulations. We clarify that clinical data is inherently noisy; by using MC Dropout during inference, our model quantifies predictive uncertainty, allowing it to flag low-confidence cases (18% in our study) for human review. This probabilistic approach prevents the model from overconfidently asserting a diagnosis in ambiguous cases.

**Location of Revision:** The mathematical justification is located in the Revised Manuscript under Section 3, "Methodology," in Subsection 3.3.1, "Uncertainty-Aware Prediction," specifically in paragraphs 2 and 3. The new text is highlighted in yellow.

**6. Figure 2 has all data equal to either 40 or 10. The authors may want to present this data in a better way.**

**Our Response:** Figure 2 has been redesigned to better illustrate the variance in the dataset. The new visualization utilizes a grouped bar chart with precise frequency counts, demonstrating the heterogeneity required for training a robust clinical classifier rather than a static distribution.

**Location of Revision:** The redesigned Figure 2 and its updated caption are located in the Revised Manuscript under Section 4, "Experimental Results," in Subsection 4.1, "Dataset Characteristics." The figure is highlighted in yellow.

**7. The tables in this paper are in different format. Please use a single format for all tables.**
**Our Response:** We have standardized all tables throughout the manuscript. Every table now utilizes the standard "three-line" publication style, with all internal vertical lines removed and consistent header alignment applied to ensure visual coherence.

**Location of Revision:** This standardization is applied throughout the Revised Manuscript to Tables 1, 4, 5, and 6. All tables are highlighted in yellow in the marked-up manuscript.

**8. Figures 4-6, 8, 11: Please rescale the y-axis to present the data variation better.**
**Our Response:** In response to this request, we have rescaled the y-axes for the performance visualizations (now labeled Figures 5, 6, 7, 9, and 12). By narrowing the y-axis range to the active performance window (e.g., 0.95–1.00), the incremental variations and convergence behaviors are now clearly discernible, confirming model stability.

**Location of Revision:** These rescaled figures are located in the Revised Manuscript under Section 5, "Results Discussion," in Subsections 5.6.3 through 5.6.8. The revised figures are highlighted in yellow.

**9. Please provide a section or paragraph to address the limitations of this study.**

**Our Response:** We have introduced a formal "Limitations" subsection. This candidly addresses the reliance on the Symptom2Disease dataset, the computational overhead of Monte Carlo sampling, and the necessity for prospective clinical trials to validate real-world utility.

**Location of Revision:** This section is located in the Revised Manuscript under Section 6, "Conclusion and Future Work," in the second paragraph of Subsection 6.1, "Limitations of the Current Study." The text is highlighted in yellow.

**Reviewer #2 – Point-by-Point Responses**

**1. Abbreviations should not be in the introduction.**

**Our Response**: We have removed all non-standard abbreviations from the Introduction. All terms are now defined at first mention, and the consolidated list is provided at the end of the manuscript as requested by Reviewer #1.

**Location of Revision:** This change is located in the Revised Manuscript in Section 1, "Introduction," specifically across paragraphs. The revised text is highlighted in yellow.

**2. Title is too long.**

**Our Response**: The title has been significantly shortened to: “*CLIN-LLM: A Safety-Constrained Hybrid Framework for Clinical Diagnosis and Treatment Generation*.”

**Location of Revision:** The revised title is located on the Title Page and in the Abstract header of the Revised Manuscript and is highlighted in yellow.

**3. Tables should be standard three-line table.**

**Our Response**: As requested, all tables (Tables 1-6) have been reformatted to conform to the standard three-line format, ensuring professional aesthetics and readability.

**Location of Revision:** This reformatting is applied throughout the Revised Manuscript to all tables, which are highlighted in yellow in the marked-up document.

**4. Algorithms should be a flow chart, not the source code in paper.**

**Our Response**: We have replaced all pseudocode and source code blocks with high-resolution, professionally rendered flowcharts (Figures 3 and 7). Figure 3 now illustrates the diagnostic reasoning logic, while Figure 7 details the retrieval-augmented treatment generation process.

**Location of Revision:** These flowcharts are located in the Revised Manuscript under Section 3, "Methodology," in Subsections 3.2 and 3.3. They are highlighted in yellow.

**5. 4.3 Implementation and Training Evaluation Metrics Precision Recall and so on should be numbered.**

**Our Response:** We have updated Section 4.3 to include hierarchical numbering for all evaluation metrics. Precision, Recall, F1-Score, and Accuracy are now clearly enumerated as 4.3.1 through 4.3.4.

**Location of Revision:** These changes are located in the Revised Manuscript under Section 4.3, "Evaluation Metrics." The new numbering is highlighted in yellow.

**6. Table 4. is a figure not a table.**

**Our Response:** We have corrected this labeling error. The qualitative case analysis (formerly Table 4) has been reclassified as Fig. 4, and subsequent items have been renumbered accordingly.

**Location of Revision:** This reclassification is located in the Revised Manuscript under Section 5.7, "Case Study Analysis." The figure is highlighted in yellow.

**7. Performance Analysis on Datasets Clinical Implications and Future Work Model Accuracy and Loss Evaluation Precision Confidence Curve should be numbered.**

**Our Response**: The manuscript's organizational structure has been revised to include a consistent numerical hierarchy. All major sections (1-6) and their corresponding subsections (e.g., 5.1, 5.2) are now numbered sequentially.

**Location of Revision:** This numerical hierarchy is applied throughout the entire Revised Manuscript from the Introduction (Section 1) to the Conclusion (Section 6). All headings are highlighted in yellow.

**8. fig 4-6 should be improved with the curves.**

**Our Response:** Figures 4, 5, and 6 (now Figures 5, 6, and 7) have been improved by replacing stepped plots with smoothed spline curves and adding data markers at 5-epoch intervals for clearer reference.

**Location of Revision:** These improved figures are located in the Revised Manuscript under Section 5, "Results Discussion." They are highlighted in yellow.

**9. What is the highlights in this paper.**

**Our Response:** We have added a distinct "Core Contributions" subsection to the Introduction, summarizing our three primary advancements: hybrid safety-constrained architecture, RxNorm integration, and uncertainty quantification.

**Location of Revision:** This new section is located in the Revised Manuscript under Section 1, "Introduction," in Subsection 1.3, "Core Contributions." The text is highlighted in yellow.

**10. Abstracts and conclusions should be improved with more highlights.**

**Our Response:** The Abstract and Conclusion have been substantially rewritten to foreground the "Safety-Constrained" nature of the framework and its impact on reducing diagnostic risk and hallucination.

**Location of Revision:** These improvements are located in the Revised Manuscript under "Abstract" (Page 1) and Section 6, "Conclusion and Future Work." Both sections are highlighted in yellow.

All the changes are highlighted using the Yellow color in the revised manuscript.
