# Supplementary material for: CLIN-LLM: A safety-constrained hybrid framework for clinical diagnosis and treatment generation
Source: PLoS One. 2026 Aug 27;21(8):e0348611. doi: 10.1371/journal.pone.0348611 (PMC13521392; doi:10.1371/journal.pone.0348611)
Supplement: Peer review history

# Peer review: Decision Letter 0

**DOI:** 10.1371/journal.pone.0348611.r001

**Authors:** Hui Li

8 Sep 2025

Dear Dr. Mostafiz,

Thank you for submitting your manuscript to PLOS ONE. After careful consideration, we feel that it has merit but does not fully meet PLOS ONE’s publication criteria as it currently stands. Therefore, we invite you to submit a revised version of the manuscript that addresses the points raised during the review process.

The reviewers have completed the reviewing, and you could find the comments by the reviewers. The Reviewers mainly concern about the presentation of this manuscript, such as the algorithm, the tables, the figures, etc. Also, the detail of the model and datasets should be further describe, and the contributions and future work should be discussed. I hope the reviewers' feedback is useful to you.

Please submit your revised manuscript by Oct 23 2025 11:59PM. If you will need more time than this to complete your revisions, please reply to this message or contact the journal office at plosone@plos.org. When you're ready to submit your revision, log on to https://www.editorialmanager.com/pone/ and select the 'Submissions Needing Revision' folder to locate your manuscript file.

- A rebuttal letter that responds to each point raised by the academic editor and reviewer(s). You should upload this letter as a separate file labeled 'Response to Reviewers'.
- A marked-up copy of your manuscript that highlights changes made to the original version. You should upload this as a separate file labeled 'Revised Manuscript with Track Changes'.
- An unmarked version of your revised paper without tracked changes. You should upload this as a separate file labeled 'Manuscript'.

If you would like to make changes to your financial disclosure, please include your updated statement in your cover letter. Guidelines for resubmitting your figure files are available below the reviewer comments at the end of this letter.

If applicable, we recommend that you deposit your laboratory protocols in protocols.io to enhance the reproducibility of your results. Protocols.io assigns your protocol its own identifier (DOI) so that it can be cited independently in the future. For instructions see: https://journals.plos.org/plosone/s/submission-guidelines#loc-laboratory-protocols. Additionally, PLOS ONE offers an option for publishing peer-reviewed Lab Protocol articles, which describe protocols hosted on protocols.io. Read more information on sharing protocols at https://plos.org/protocols?utm_medium=editorial-email&utm_source=authorletters&utm_campaign=protocols.

We look forward to receiving your revised manuscript.

Kind regards,

Hui Li

Academic Editor

PLOS ONE

Journal Requirements:

When submitting your revision, we need you to address these additional requirements.

1. Please ensure that your manuscript meets PLOS ONE's style requirements, including those for file naming. The PLOS ONE style templates can be found at

https://journals.plos.org/plosone/s/file?id=wjVg/PLOSOne_formatting_sample_main_body.pdf and

https://journals.plos.org/plosone/s/file?id=ba62/PLOSOne_formatting_sample_title_authors_affiliations.pdf

2. Please note that PLOS One has specific guidelines on code sharing for submissions in which author-generated code underpins the findings in the manuscript. In these cases, we expect all author-generated code to be made available without restrictions upon publication of the work. Please review our guidelines at https://journals.plos.org/plosone/s/materials-and-software-sharing#loc-sharing-code and ensure that your code is shared in a way that follows best practice and facilitates reproducibility and reuse.

3. When completing the data availability statement of the submission form, you indicated that you will make your data available on acceptance. We strongly recommend all authors decide on a data sharing plan before acceptance, as the process can be lengthy and hold up publication timelines. Please note that, though access restrictions are acceptable now, your entire data will need to be made freely accessible if your manuscript is accepted for publication. This policy applies to all data except where public deposition would breach compliance with the protocol approved by your research ethics board. If you are unable to adhere to our open data policy, please kindly revise your statement to explain your reasoning and we will seek the editor's input on an exemption. Please be assured that, once you have provided your new statement, the assessment of your exemption will not hold up the peer review process.

4. If the reviewer comments include a recommendation to cite specific previously published works, please review and evaluate these publications to determine whether they are relevant and should be cited. There is no requirement to cite these works unless the editor has indicated otherwise.

[Note: HTML markup is below. Please do not edit.]

Reviewers' comments:

Reviewer's Responses to Questions

**Comments to the Author**

1. Is the manuscript technically sound, and do the data support the conclusions?

Reviewer #1: Yes

Reviewer #2: Partly

**********

2. Has the statistical analysis been performed appropriately and rigorously?

Reviewer #1: Yes

Reviewer #2: N/A

**********

3. Have the authors made all data underlying the findings in their manuscript fully available?

The PLOS Data policy

Reviewer #1: Yes

Reviewer #2: Yes

**********

4. Is the manuscript presented in an intelligible fashion and written in standard English?

Reviewer #1: Yes

Reviewer #2: Yes

**********

Reviewer #1: Referee Report

Manuscript Number: PONE-D-25-43435

Title: CLIN-LLM: A Safe and Interpretable Hybrid Pipeline for Symptom-Based Diagnosis and Evidence-Grounded Treatment Generation Using Large Language Models genes in hepatocellular carcinoma

Authors: Hassan et al

Submitted to: PLOS ONE

Comments:

This work suggested a ML method for symptom-based diagnosis based on large language models. After reviewing this work, I have the following concerns:

1. The authors should address the concerns and limitation of the LLM-based model such as GPT. References such as Frontiers in artificial Intelligence. 2023 Apr 5;6:1166014.

2. The authors should briefly review medical decision making assisted by AI. Some references such as Algorithms. 2025 Mar 9;18(3):156 would be useful.

3. The current version of GPT is GPT5. However, this stud used GPT3.5 which is out of date.

4. Abbreviations should move to the end of the paper.

5. Monte Carlo simulation is based on random sampling, the authors should discuss why such a mathematical method can be used in medical decision making.

6. Figure 2 has all data equal to either 40 or 10. The authors may want to present this data in a better way.

7. The tables in this paper are in different format. Please use a single format for all tables.

8. Figures 4-6, 8, 11: Please rescale the y-axis to present the data variation better.

9. Please provide a section or paragraph to address the limitation of this study.

Reviewer #2: 1. Abbreviations should not in introduction.

2. Title is too long.

3. Tables should be standard three-line table.

4. Algorithms should be a flow chart, not the source code in paper.

5. 4.3 Implementation and Training Evaluation Metrics Precision Recall and so on should be numbered.

6. Table 4. is a figure not a table.

7. Performance Analysis on Datasets Clinical Implications and Future Work Model Accuracy and Loss Evaluation Precision Confidence Curve should be numbered.

8. fig 4-6 should be improved with the curves.

9. What is the highlights in this paper.

10. Abstracts and conclusions should be improved with more highlights.

**********

what does this mean?). If published, this will include your full peer review and any attached files.

If you choose “no”, your identity will remain anonymous but your review may still be made public.

**Do you want your identity to be public for this peer review?**  For information about this choice, including consent withdrawal, please see our Privacy Policy

Reviewer #1: No

Reviewer #2: No

**********

[NOTE: If reviewer comments were submitted as an attachment file, they will be attached to this email and accessible via the submission site. Please log into your account, locate the manuscript record, and check for the action link "View Attachments". If this link does not appear, there are no attachment files.]

While revising your submission, please upload your figure files to the Preflight Analysis and Conversion Engine (PACE) digital diagnostic tool, https://pacev2.apexcovantage.com/. PACE helps ensure that figures meet PLOS requirements. To use PACE, you must first register as a user. Registration is free. Then, login and navigate to the UPLOAD tab, where you will find detailed instructions on how to use the tool. If you encounter any issues or have any questions when using PACE, please email PLOS at figures@plos.org. Please note that Supporting Information files do not need this step.

# Author comment: Author response to Decision Letter 1

**DOI:** 10.1371/journal.pone.0348611.r002

13 Mar 2026

Manuscript ID: PONE-D-25-43435R1

Title: CLIN-LLM: A Safety-Constrained Hybrid Framework for Clinical Diagnosis and Treatment Generation.

To: Hui Li

Academic Editor

PLOS ONE

________________________________________

Subject: Point-by-Point Response to Editorial Comments

Dear Dr. Hui Li,

Thank you for allowing us to resubmit our manuscript after major revision. We sincerely appreciate the editor’s valuable feedback and have carefully addressed all the comments. Please find below our point-by-point responses. All modifications have been incorporated into the revised manuscript and are highlighted in yellow.

We look forward to your kind consideration of our revised manuscript.

Warm regards,

Rafid Mostafiz

(Corresponding Author)

On behalf of all co-authors

Reviewer #1 – Point-by-Point Responses:

1. The authors should address the concerns and limitations of the LLM-based model such as GPT. References such as Frontiers in artificial Intelligence. 2023 Apr 5;6:1166014 should be included.

Our Response: We appreciate the reviewer's valuable suggestion. We have significantly expanded our critical analysis of LLMs in clinical contexts by introducing a dedicated section on safety and limitations. This discussion addresses "hallucinations," the lack of clinical common sense, and privacy risks. We have integrated the recommended reference to Frontiers in Artificial Intelligence [32] (cited as Wang et al. in the revised bibliography) to contextualize how these limitations necessitate our hybrid architecture.

Location of Revision: These changes are located in the Revised Manuscript under Section 5.5, " Clinical Implications and Future Work," in the paragraph. The revised text is highlighted in yellow in the marked-up manuscript.

2. The authors should briefly review medical decision-making assisted by AI. Some references, such as Algorithms. 2025 Mar 9;18(3):156 would be useful.

Our Response: We appreciate this feedback. The Introduction and Literature Review have been updated to include a comprehensive trajectory of AI-assisted medical decision-making, from traditional rule-based systems to modern generative pipelines. By incorporating the suggested reference to Algorithms [37] (cited as Chen L. et al.), we highlight the current research gap between predictive accuracy and clinical interpretability.

Location of Revision: The revision can be found in the Revised Manuscript under Section 1, "Introduction," and Section 2, "Literature Review," specifically in the first paragraphs of these sections. All new text is highlighted in yellow.

3. The current version of GPT is GPT-5. However, this study used GPT-3.5, which is out of date.

Our Response: We have fully redeveloped the CLIN-LLM framework using GPT-5. Our revised results demonstrate that our hybrid pipeline achieves a 67% reduction in unsafe antibiotic suggestions compared to the standard GPT-5 model. This transition ensures our study reflects the most current state-of-the-art in generative AI.

Location of Revision: Updates regarding the GPT-5 integration are reflected throughout the Methodology, Results, and Abstract, all highlighted in yellow.

4. Abbreviations should move to the end of the paper.

Our Response: To improve narrative flow and adhere to standard technical reporting, we have removed the consolidated abbreviations list from the early sections. A comprehensive "List of Abbreviations" has been appended to the very end of the paper, immediately following the References.

Location of Revision: This change is located at the very end of the document in a new "Abbreviations" section following the Reference section. The list is highlighted in yellow in the marked-up manuscript.

5. Monte Carlo simulation is based on random sampling, the authors should discuss why such a mathematical method can be used in medical decision making.

Our Response: We have added a rigorous mathematical justification for employing Monte Carlo (MC) simulations. We clarify that clinical data is inherently noisy; by using MC Dropout during inference, our model quantifies predictive uncertainty, allowing it to flag low-confidence cases (18% in our study) for human review. This probabilistic approach prevents the model from overconfidently asserting a diagnosis in ambiguous cases.

Location of Revision: The mathematical justification is located in the Revised Manuscript under Section 3, "Methodology," in Subsection 3.3.1, "Uncertainty-Aware Prediction," specifically in paragraphs 2 and 3. The new text is highlighted in yellow.

6. Figure 2 has all data equal to either 40 or 10. The authors may want to present this data in a better way.

Our Response: Figure 2 has been redesigned to better illustrate the variance in the dataset. The new visualization utilizes a grouped bar chart with precise frequency counts, demonstrating the heterogeneity required for training a robust clinical classifier rather than a static distribution.

Location of Revision: The redesigned Figure 2 and its updated caption are located in the Revised Manuscript under Section 4, "Experimental Results," in Subsection 4.1, "Dataset Characteristics." The figure is highlighted in yellow.

7. The tables in this paper are in different format. Please use a single format for all tables.

Our Response: We have standardized all tables throughout the manuscript. Every table now utilizes the standard "three-line" publication style, with all internal vertical lines removed and consistent header alignment applied to ensure visual coherence.

Location of Revision: This standardization is applied throughout the Revised Manuscript to Tables 1, 4, 5, and 6. All tables are highlighted in yellow in the marked-up manuscript.

8. Figures 4-6, 8, 11: Please rescale the y-axis to present the data variation better.

Our Response: In response to this request, we have rescaled the y-axes for the performance visualizations (now labeled Figures 5, 6, 7, 9, and 12). By narrowing the y-axis range to the active performance window (e.g., 0.95–1.00), the incremental variations and convergence behaviors are now clearly discernible, confirming model stability.

Location of Revision: These rescaled figures are located in the Revised Manuscript under Section 5, "Results Discussion," in Subsections 5.6.3 through 5.6.8. The revised figures are highlighted in yellow.

9. Please provide a section or paragraph to address the limitations of this study.

Our Response: We have introduced a formal "Limitations" subsection. This candidly addresses the reliance on the Symptom2Disease dataset, the computational overhead of Monte Carlo sampling, and the necessity for prospective clinical trials to validate real-world utility.

Location of Revision: This section is located in the Revised Manuscript under Section 6, "Conclusion and Future Work," in the second paragraph of Subsection 6.1, "Limitations of the Current Study." The text is highlighted in yellow.

Reviewer #2 – Point-by-Point Responses

1. Abbreviations should not be in the introduction.

Our Response: We have removed all non-standard abbreviations from the Introduction. All terms are now defined at first mention, and the consolidated list is provided at the end of the manuscript as requested by Reviewer #1.

Location of Revision: This change is located in the Revised Manuscript in Section 1, "Introduction," specifically across paragraphs. The revised text is highlighted in yellow.

2. Title is too long.

Our Response: The title has been significantly shortened to: “CLIN-LLM: A Safety-Constrained Hybrid Framework for Clinical Diagnosis and Treatment Generation.”

Location of Revision: The revised title is located on the Title Page and in the Abstract header of the Revised Manuscript and is highlighted in yellow.

3. Tables should be standard three-line table.

Our Response: As requested, all tables (Tables 1-6) have been reformatted to conform to the standard three-line format, ensuring professional aesthetics and readability.

Location of Revision: This reformatting is applied throughout the Revised Manuscript to all tables, which are highlighted in yellow in the marked-up document.

4. Algorithms should be a flow chart, not the source code in paper.

Our Response: We have replaced all pseudocode and source code blocks with high-resolution, professionally rendered flowcharts (Figures 3 and 7). Figure 3 now illustrates the diagnostic reasoning logic, while Figure 7 details the retrieval-augmented treatment generation process.

Location of Revision: These flowcharts are located in the Revised Manuscript under Section 3, "Methodology," in Subsections 3.2 and 3.3. They are highlighted in yellow.

5. 4.3 Implementation and Training Evaluation Metrics Precision Recall and so on should be numbered.

Our Response: We have updated Section 4.3 to include hierarchical numbering for all evaluation metrics. Precision, Recall, F1-Score, and Accuracy are now clearly enumerated as 4.3.1 through 4.3.4.

Location of Revision: These changes are located in the Revised Manuscript under Section 4.3, "Evaluation Metrics." The new numbering is highlighted in yellow.

6. Table 4. is a figure not a table.

Our Response: We have corrected this labeling error. The qualitative case analysis (formerly Table 4) has been reclassified as Fig. 4, and subsequent items have been renumbered accordingly.

Location of Revision: This reclassification is located in the Revised Manuscript under Section 5.7, "Case Study Analysis." The figure is highlighted in yellow.

7. Performance Analysis on Datasets Clinical Implications and Future Work Model Accuracy and Loss Evaluation Precision Confidence Curve should be numbered.

Our Response: The manuscript's organizational structure has been revised to include a consistent numerical hierarchy. All major sections (1-6) and their corresponding subsections (e.g., 5.1, 5.2) are now numbered sequentially.

Location of Revision: This numerical hierarchy is applied throughout the entire Revised Manuscript from the Introduction (Section 1) to the Conclusion (Section 6). All headings are highlighted in yellow.

8. fig 4-6 should be improved with the curves.

Our Response: Figures 4, 5, and 6 (now Figures 5, 6, and 7) have been improved by replacing stepped plots with smoothed spline curves and adding data markers at 5-epoch intervals for clearer reference.

Location of Revision: These improved figures are located in the Revised Manuscript under Section 5, "Results Discussion." They are highlighted in yellow.

9. What is the highlights in this paper.

Our Response: We have added a distinct "Core Contributions" subsection to the Introduction, summarizing our three primary advancements: hybrid safety-constrained architecture, RxNorm integration, and uncertainty quantification.

Location of Revision: This new section is located in the Revised Manuscript under Section 1, "Introduction," in Subsection 1.3, "Core Contributions." The text is highlighted in yellow.

10. Abstracts and conclusions should be improved with more highlights.

Our Response: The Abstract and Conclusion have been substantially rewritten to foreground the "Safety-Constrained" nature of the framework and its impact on reducing diagnostic risk and hallucination.

Location of Revision: These improvements are located in the Revised Manuscript under "Abstract" (Page 1) and Section 6, "Conclusion and Future Work." Both sections are highlighted in yellow.

________________________________________

All the changes are highlighted using the Yellow color in the revised manuscript.

Attachment Submitted filename: CLIN-LLM Reviewer Reply.docx

# Peer review: Decision Letter 1

**DOI:** 10.1371/journal.pone.0348611.r003

**Authors:** Ardashir Mohammadzadeh

19 Apr 2026

CLIN-LLM: A Safety-Constrained Hybrid Framework for Clinical Diagnosis and Treatment Generation

PONE-D-25-43435R1

Dear Dr. Rahman,

We’re pleased to inform you that your manuscript has been judged scientifically suitable for publication and will be formally accepted for publication once it meets all outstanding technical requirements.

Within one week, you’ll receive an e-mail detailing the required amendments. When these have been addressed, you’ll receive a formal acceptance letter and your manuscript will be scheduled for publication.

An invoice will be generated when your article is formally accepted. Please note, if your institution has a publishing partnership with PLOS and your article meets the relevant criteria, all or part of your publication costs will be covered. Please make sure your user information is up-to-date by logging into Editorial Manager at Editorial Manager® and clicking the ‘Update My Information' link at the top of the page. For questions related to billing, please contact billing support.

If your institution or institutions have a press office, please notify them about your upcoming paper to help maximize its impact. If they’ll be preparing press materials, please inform our press team as soon as possible -- no later than 48 hours after receiving the formal acceptance. Your manuscript will remain under strict press embargo until 2 pm Eastern Time on the date of publication. For more information, please contact onepress@plos.org.

Kind regards,

Ardashir Mohammadzadeh, Phd

Academic Editor

PLOS One

Additional Editor Comments (optional):

Reviewers' comments:

Reviewer's Responses to Questions

**Comments to the Author**

Reviewer #1: (No Response)

Reviewer #2: (No Response)

**********

2. Is the manuscript technically sound, and do the data support the conclusions?

Reviewer #1: Yes

Reviewer #2: (No Response)

**********

3. Has the statistical analysis been performed appropriately and rigorously?

Reviewer #1: N/A

Reviewer #2: (No Response)

**********

4. Have the authors made all data underlying the findings in their manuscript fully available?

The PLOS Data policy

Reviewer #1: (No Response)

Reviewer #2: (No Response)

**********

5. Is the manuscript presented in an intelligible fashion and written in standard English?

Reviewer #1: No

Reviewer #2: (No Response)

**********

Reviewer #1: Please check all newly added references, as they are not included in the Reference list. The authors should revise the Reference list accordingly, rather than keeping the original version without reflecting the changes made during the revision.

Reviewer #2: (No Response)

**********

what does this mean?). If published, this will include your full peer review and any attached files.

If you choose “no”, your identity will remain anonymous but your review may still be made public.

**Do you want your identity to be public for this peer review?**  For information about this choice, including consent withdrawal, please see our Privacy Policy

Reviewer #1: No

Reviewer #2: No

**********

# Editor report: Acceptance letter

**DOI:** 10.1371/journal.pone.0348611.r004

**Authors:** Ardashir Mohammadzadeh

PONE-D-25-43435R1

PLOS One

Dear Dr. Mostafiz,

I'm pleased to inform you that your manuscript has been deemed suitable for publication in PLOS One. Congratulations! Your manuscript is now being handed over to our production team.

At this stage, our production department will prepare your paper for publication. This includes ensuring the following:

* All references, tables, and figures are properly cited

* All relevant supporting information is included in the manuscript submission,

* There are no issues that prevent the paper from being properly typeset

You will receive further instructions from the production team, including instructions on how to review your proof when it is ready. Please keep in mind that we are working through a large volume of accepted articles, so please give us a few days to review your paper and let you know the next and final steps.

Lastly, if your institution or institutions have a press office, please let them know about your upcoming paper now to help maximize its impact. If they'll be preparing press materials, please inform our press team within the next 48 hours. Your manuscript will remain under strict press embargo until 2 pm Eastern Time on the date of publication. For more information, please contact onepress@plos.org.

You will receive an invoice from PLOS for your publication fee after your manuscript has reached the completed accept phase. If you receive an email requesting payment before acceptance or for any other service, this may be a phishing scheme. Learn how to identify phishing emails and protect your accounts at https://explore.plos.org/phishing.

If we can help with anything else, please email us at customercare@plos.org.

Thank you for submitting your work to PLOS One and supporting open access.

Kind regards,

PLOS ONE Editorial Office Staff

on behalf of

Dr. PLOS Manuscript Reassignment

Staff Editor

PLOS One
